# Surgical myectomy for hypertrophic cardiomyopathy: procedural volume and outcomes

**DOI:** 10.1093/eurheartj/ehaf560

**Published:** 2025-08-29

**Authors:** Tijn J P Heeringa, Romy M J J Hegeman, Yvonne Koop, Sulayman el Mathari, Marieke Hoogewerf, Maaike M Roefs, Karin C Smits, Dorien Laenens, Giulia De Zan, Maarten J Cramer, Marco Guglielmo, Pim van der Harst, Ilonca Vaartjes, Mostafa M Mokhles, Patrick Klein, Niels P van der Kaaij

**Affiliations:** Department of Cardiothoracic Surgery, University Medical Centre Utrecht, 3584 CX, Heidelberglaan 100, Utrecht, The Netherlands; Julius Centre for Health Sciences and Primary Care, Cardiovascular Epidemiology, University Medical Centre Utrecht, Utrecht University, Utrecht, The Netherlands; Department of Cardiothoracic Surgery, Sint Antonius Hospital Nieuwegein, Niewegein, The Netherlands; Department of Cardiothoracic Surgery, Amsterdam University Medical Centre, Amsterdam, The Netherlands; Julius Centre for Health Sciences and Primary Care, Cardiovascular Epidemiology, University Medical Centre Utrecht, Utrecht University, Utrecht, The Netherlands; Department of Research, Dutch Heart Foundation, The Hague, The Netherlands; Department of Cardiothoracic Surgery, Amsterdam University Medical Centre, Amsterdam, The Netherlands; Department of Cardiothoracic Surgery, Erasmus University Medical Centre, Dr. Molenwaterplein 40, Rotterdam 3015 GD, The Netherlands; Department of Cardiology, University Medical Centre Utrecht, Utrecht, the Netherlands; Department of Research, Netherlands Heart Registration, Utrecht, The Netherlands; Department of Cardiothoracic Surgery, Thorax Centrum Twente, Medisch Spectrum Twente, The Netherlands; Department of Physiology, Maastricht University Medical Centre+, Cardiovascular Research Institute Maastricht, Maastricht, The Netherlands; Department of Cardiology, Leiden University Medical Centre, Leiden, The Netherlands; Department of Cardiology, University Medical Centre Utrecht, Utrecht, the Netherlands; Department of Cardiology, University Medical Centre Utrecht, Utrecht, the Netherlands; Department of Cardiology, University Medical Centre Utrecht, Utrecht, the Netherlands; Department of Cardiology, University Medical Centre Utrecht, Utrecht, the Netherlands; Julius Centre for Health Sciences and Primary Care, Cardiovascular Epidemiology, University Medical Centre Utrecht, Utrecht University, Utrecht, The Netherlands; Department of Cardiothoracic Surgery, University Medical Centre Utrecht, 3584 CX, Heidelberglaan 100, Utrecht, The Netherlands; Department of Cardiothoracic Surgery, Sint Antonius Hospital Nieuwegein, Niewegein, The Netherlands; Department of Cardiothoracic Surgery, Amsterdam University Medical Centre, Amsterdam, The Netherlands; Department of Cardiothoracic Surgery, University Medical Centre Utrecht, 3584 CX, Heidelberglaan 100, Utrecht, The Netherlands; Department of Cardiothoracic Surgery, Erasmus University Medical Centre, Dr. Molenwaterplein 40, Rotterdam 3015 GD, The Netherlands

**Keywords:** Hypertrophic obstructive cardiomyopathy, Heart septum, Surgical volume, Morrow, Echocardiography, Left ventricular outflow obstruction

## Abstract

**Background and Aims:**

Outcomes after surgical myectomy in hypertrophic obstructive cardiomyopathy (HOCM) patients have not been investigated in a multicentre registry with detailed clinical data. Hence, the objectives of this multicentre Dutch study are to describe the 30-day clinical outcomes after surgical myectomy, and to assess factors associated with increased 30-day complication rates.

**Methods:**

All HOCM patients (*n* = 335) who underwent surgery between 2012 and 2020 across 12 Dutch hospitals were analysed using data from the Netherlands Heart Registration. Multiple logistic regression analyses assessed factors associated with a higher 30-day complication rate.

**Results:**

Isolated surgical myectomy was performed in 22%, surgical myectomy with one concomitant procedure in 54%, and surgical myectomy with two or more concomitant procedures in 24%. Thirty-day complication rates concerned mortality (5%), ventricular septal defect (2%), stroke (3%), and surgical reoperation (2%). Mean resting left ventricular outflow tract (LVOT) gradient improved from 61 ± 30 mmHg to 13 ± 12 mmHg postoperatively, systolic anterior motion from 80% to 8%, and mitral regurgitation grade 3 or 4 from 31% to 6%. Low-volume hospital (<10 surgical myectomy procedures/year), female sex, and ≥2 concomitant procedures were significantly associated with increased 30-day complication rates. The adjusted 30-day complication rate was increased in low-volume hospitals (odds ratio 3.23 (95% confidence interval: 1.43–8.09); *P* = .007).

**Conclusions:**

Surgical myectomy with or without concomitant procedures effectively relieved LVOT obstruction in 93% of patients in this multicentre cohort. Female sex, ≥ 2 concomitant procedures, and low-volume hospitals were associated with higher 30-day complication rates. Although an inverse volume–complication relation was observed, this finding should be interpreted cautiously, and further investigation in larger sample size studies is warranted.

## Structured Graphical Abstract

**Structured Graphical Abstract:**
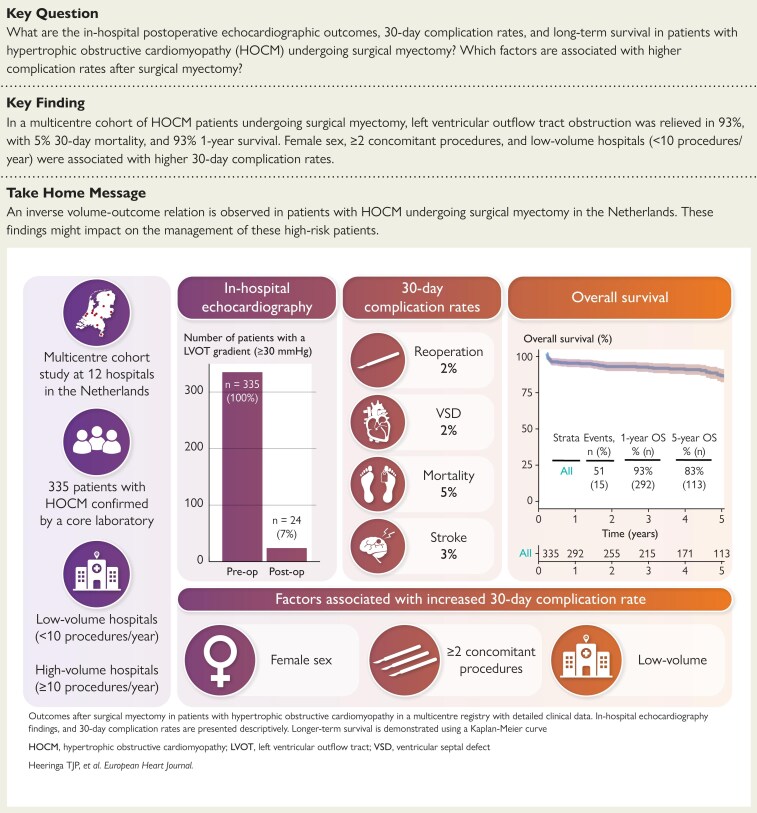

**See the editorial comment for this article ‘The dilemma of ‘real-world’ (low-volume) surgical myectomy for symptomatic obstructive hypertrophic cardiomyopathy’, by B.J. Maron *et al.*, https://doi.org/10.1093/eurheartj/ehaf561.**

## Introduction

Hypertrophic cardiomyopathy (HCM) is a common genetically inherited myocardial disease.^1^ HCM has an estimated incidence between 1 in 200 to 1 in 500 in the general population and often involves dynamic left ventricular outflow tract (LVOT) obstruction.^1,2^ Resting or provoked LVOT obstruction with a gradient exceeding 30 mmHg is referred to as hypertrophic obstructive cardiomyopathy (HOCM).^1^ In patients with HOCM where medication treatment is insufficient, invasive options such as surgical myectomy (surgical myectomy) or septum alcohol ablation may alleviate LVOT obstruction.^1^ Among these options, the surgical myectomy procedure as developed by Morrow *et al*. remains the gold standard procedure to treat LVOT obstruction.^3,4^

The surgical procedure significantly reduces the LVOT pressure gradient in over 90% of patients, enhances quality of life, and improves New York Heart Association (NYHA) functional class by at least one class.^5^ Moreover, complication rates associated with the procedure are low, though they can be severe. Complications include mortality (< 1%), ventricular septal defect (VSD; 1%), remaining LVOT obstruction requiring surgical reoperation (< 3%), stroke (< 2%), complete heart block (2%), and aortic regurgitation (< 1%).^1,6–10^ These complication rates after surgical myectomy have mostly been investigated in single-centre studies from hospitals with large patient series, while hospitals with less experience in performing surgical myectomy procedures tend to report higher complication rates.^11^ At the same time, recent multicentre studies in the United States presented an inverse relationship between hospital procedural volume and surgical myectomy outcomes.^11–14^ In this regard, hospitals performing 10 or more surgical myectomy procedures per year had lower mortality rates and incidence of VSD than hospitals with lower procedural volumes.^13^ The 2023 European Society of Cardiology guidelines recommends that HOCM patients who are candidates for surgical myectomy be referred to experienced operators within a multidisciplinary team specialized in the management of HCM (Class IC recommendation).^1^ However, this guideline has not provided a procedural volume cut-off for hospitals performing surgical myectomy.

Although these recent studies in the United States included large sample sizes, they lacked echocardiographic data, did not take into account important confounders for outcomes like concomitant procedures nor investigated the incidence of surgical reoperations.^11–14^ To address these limitations, the objectives of this multicentre Dutch study are to describe 30-day clinical outcomes using complications and echocardiography data after surgical myectomy, and to assess which factors are associated with increased complication rates with a follow-up period of 30 days. In addition, the authors will report longer-term overall survival.

## Methods

### Design, setting, and study population

Patients who underwent surgical myectomy were identified from the Netherlands Heart Registration (NHR) database, which is a prospective database that contains data on all cardiac surgical interventions performed in the Netherlands, including information on preoperative patient characteristics, intervention variables, and clinical outcomes.^15^ Multiple quality checks have been performed on the data during the data collection and cardiac hospitals have been audited annually.^16^ This study was a multicentre cohort study conducted with participation from 12 out of 14 cardiothoracic surgery hospitals (86%) performing surgical myectomy procedures in the Netherlands (members of the Cardiothoracic Surgery Registration Committee of the NHR and members of the study group of the different hospitals are listed in the Supplementary material, *Table S1* and *Table S2*). Patients were included if they underwent a surgical myectomy procedure between January 2012 and December 2020 and were ≥18 years old at the time of the procedure. Diagnostic verification of HCM was performed by a core laboratory of two experienced imaging cardiologists (M.G. and M.J.C.) using electronic health records and echocardiography images. Diagnostic criteria were in accordance with the 2020 AHA/ACC guidelines; myocardial end-diastolic wall thickness is ≥ 15 mm in any myocardial segment that is not explained by loading conditions.^17–20^ Echocardiographic images from two different time points—preoperative, and the first postoperative echocardiography—were re-evaluated. They were assessed according to the recommendation of the European Association of Cardiovascular Imaging and recommendations from the American Society of Echocardiography.^18,20^ Approval from the Institutional Review Boards of 12 hospitals was obtained for this study (the Review Board Approvals are listed in Supplementary data online, *Table S3*).

### Patient and treatment characteristics

Electronic health records of all included patients were manually reviewed to verify, complete, and adjust the NHR data when needed (see Supplementary material, *Data S1* ∼ routine NHR variables). Mortality data were derived from the Dutch Population Registration, which is an accurate and up-to-date governmental database containing information about all residents of the Netherlands. In addition to routine NHR data, additional data were collected on patient characteristics, echocardiography, surgical procedure, and follow-up variables (see Supplementary material, *Data S1* ∼ additional NHR variables). We requested cardiology outpatient reports, ultrasound reports, and echocardiographic DICOM files from the outpatient clinic or the referring hospital if the reports or DICOM files were unavailable in the hospital where the patient underwent the surgical myectomy procedure. A timeline for data collection is provided in the Supplementary data online, *Figure S1*.

Surgical myectomy was categorized into isolated surgical myectomy, surgical myectomy with one concomitant procedure [coronary artery bypass grafting (CABG), mitral valve repair or replacement, aortic valve replacement (AVR), tricuspid valve surgery, pulmonary valve surgery, aortic surgery, and rhythm surgery], and surgical myectomy with two or more concomitant procedures. Procedures that were conceivably the result of operative complications were not considered concomitant procedures; these include implantable cardiac defibrillator (ICD) insertion, permanent pacemaker implantation, and VSD repair.

### Primary outcome

The primary outcome measures included 30-day complication rates after surgical myectomy and the first postoperative echocardiographic measurements. The complications recorded were mortality, VSD, stroke with permanent damage, and surgical reoperation. Surgical reoperation was defined as a second surgical procedure (surgical myectomy, mitral valve repair, and/or mitral valve replacement), other than re-exploration for bleeding, tamponade, or sternum wound problems.^21^ The complication was recorded if a patient had at least one of the complications either during hospital admission or within 30 calendar days after the surgical myectomy procedure. Echocardiographic outcomes included LVOT gradient, valvular systolic anterior motion (SAM), and mitral regurgitation.^17,19^ A postoperative resting LVOT gradient of <30 mmHg or provoked LVOT gradient of <50 mmHg was defined as the effectiveness of surgical myectomy.

### Mean hospital procedural volume

Mean hospital procedural volume was calculated as the total number of surgical myectomy procedures performed at a hospital from January 2012 through December 2020 divided by the years data were collected. The cut-off of a high- vs. low-volume hospital was based on scientific literature; hospitals performing surgical myectomy are categorized as high-volume when 10 or more surgical myectomy procedures per year were performed and low-volume when <10 surgical myectomy procedures per year were performed.^11,12,22^ In the volume–outcome relationship assessment, outcome was defined as the composite of mortality, VSD, stroke, and surgical reoperation.

### Statistical methods

#### Missing values

In general, the national database of the NHR has high data completeness. After applying the exclusion criteria, missing data were observed for some variables; details are presented per variable in the Supplementary data online, *Tables S4* and *S5*. Seven variables had relatively higher levels of missing data: aortic cross-clamp (ACC) time (8%), cardiopulmonary bypass (CPB) time (8%), recurrent myocardial infarction (8%), postoperative left atrial volume index (LAVI; 10%), LAVI at baseline (10%), provocative LVOT gradient at baseline (50%), pathogenetic DNA variants (55%), and postoperative provocative LVOT gradient (80%). Therefore, multiple imputation by chained equations was performed to impute missing values. Data were imputed 50 times under fully conditional specification. Covariates with more than 50% missing values were excluded from the imputation process.^23^ The imputation model included baseline characteristics, categorized surgical procedures, and two postoperative echocardiographic measurements (postoperative SAM and postoperative resting LVOT gradient). The imputation process was assessed visually using strip plots and density plots. Analyses [i.e. Firth’s logistic regression and generalized linear mixed models (GLMMs)] were performed on each imputation, results were subsequently pooled with Rubin’s Rules. False discovery rate correction using the Benjamini–Hochberg method was applied for multiple comparisons.

#### Statistical analyses

Normally distributed continuous variables were presented as mean ± standard deviation and compared using the Student’s *t*-test. Skewed continuous variables were presented as median and interquartile range [1st to 3rd quartile] and compared using the Mann–Whitney U test. The normality of the distributions was tested by visual inspection of the quantile–quantile (Q–Q) plot. Categorical variables were presented as counts and percentages (%) and compared using the χ^2^ test or Fisher’s exact tests, as appropriate. Clopper–Pearson exact method was used to calculate the confidence intervals (CI) for proportions. A sensitivity analysis was performed for isolated surgical myectomy to understand the impact of concomitant procedures.

#### Firth’s logistic regression analyses

Factors associated with the composite 30-day complication rate were assessed with Firth’s logistic regression analyses. To construct a complete multivariable model, all pre-specified clinically relevant variables were selected. Finally, effect modification was evaluated via statistical testing of an interaction term for concomitant cardiac procedures and hospital volume to test whether the odds ratio (OR) of hospital volume was modified by the presence of concomitant cardiac procedures. A sensitivity analysis was performed to assess the impact of center-level clustering. A GLMM was used to estimate the degree of clustering (variance component of the intercept).

#### Survival analyses

The Kaplan-Meier method was used to visualize the overall survival curves and estimate the 1-year, and 5-year overall survival of patients with HOCM who underwent surgical myectomy.

All statistical analyses were performed using the computing and statistics program R (the R Foundation for Statistical Computing, Vienna, Austria, Version 4.1.2) with R package ggplot2 to construct figures. All statistical tests with a *P*-value ≤ .05 were considered significant.

## Results

The NHR database contained data on 503 patients who underwent surgical myectomy in 12 hospitals between January 2012 and December 2020. The final cohort included 335 patients from 12 participating hospitals. A total of 168 patients were excluded, primarily due to lack of informed consent (*n* = 44 [9%]), missing preoperative echocardiographic images (*n* = 56 [11%]), failure to meet the inclusion criteria (e.g. sub-valvular membrane resection (*n* = 22 [4%]), and other reasons (*n* = 46 [9%]); (Supplementary data online, *Figure S2*).

### Patient and intraoperative characteristics

Mean age at time of surgery was 62 ± 12 years, and 53% of patients were male; 61% were in NYHA functional classes III or IV, and alcohol septal ablation (ASA) was performed in 4% (see Supplementary data online, *Tables S6* and *S7*) of the patients, before surgery. The preoperative mean resting LVOT gradient was 61 ± 30 mmHg, and the mean provocative LVOT gradient was 85 ± 28 mmHg. The mean interventricular septal thickness in diastole (IVSD) was 21 ± 4 mm, valvular SAM was present in 268 (80%) patients, and mitral regurgitation grade 3 or 4 was present in 103 (31%) patients. Isolated surgical myectomy was performed in 22% (*n* = 72) of the patients, while one concomitant surgical procedure (54% [*n* = 181]), and two or more concomitant surgical procedures (24% [*n* = 82]) in the remaining. Concomitant mitral valve repair (see Supplementary data online, *Figure S3*, *Tables S10* and *S11*), concomitant mitral valve replacement, and concomitant AVR were performed in respectively 36% (*n* = 121), 16% (*n* = 52), and 22% (*n* = 75) of patients. *Tables 1* and *2* display patient and intraoperative characteristics stratified by hospital volume.

**Table 1:** Patient characteristics of HOCM patients operated on with surgical myectomy in The Netherlands stratified by high- or low-volume hospital

Characteristics	Overall (*n* = 335)	High-volume (*n* = 159)	Low-volume (*n* = 176)
Age, years, median [IQR]	65 [56–70]	65 [55–70]	66 [58–71]
Male sex, *n* (%)	179 (53)	85 (53)	94 (53)
BMI, kg/m^2^, median [IQR]	27 [24–31]	27 [24–29]	27 [24–31]
Diabetes, *n* (%)	56 (17)	22 (14)	24 (14)
Creatinine, μmol/L, median [IQR]	81 [71–96]	79 [70–93]	83 [72–97]
EuroSCORE II, median [IQR]	2 [1–3]	2 [1–3]	2 [1–3]
Prior cardiac surgery, *n* (%)	16 (5)	6 (4)	10 (6)
Prior ASA, *n* (%)	13 (4)	6 (4)	7 (4)
Chronic lung disease, *n* (%)	40 (12)	19 (12)	21 (11)
Extracardiac arteriopathy, *n* (%)	15 (4)	5 (3)	10 (6)
Neurologic dysfunction, *n* (%)	7 (2)	2 (1)	5 (3)
Atrial fibrillation history, *n* (%)	65 (19)	37 (23)	28 (16)
ICD history, *n* (%)	22 (7)	9 (6)	13 (7)
Pacemaker history, *n* (%)	9 (3)	1 (1)	8 (5)
LBBB, *n* (%)	33 (10)	15 (10)	18 (10)
RBBB, *n* (%)	24 (7)	9 (5)	15 (9)
Emergency operation, *n* (%)	5 (2)	1 (1)	4 (2)
Endocarditis, *n* (%)	3 (9)	2 (1)	1 (1)
Pathogenetic DNA variants^a,b^, *n* (%)	87 (58)	39 (71)	48 (50)
Family history of HCM (first-degree relative), *n* (%)	55 (16)	29 (18)	26 (15)
NYHA functional class, *n* (%)			
I-II	131 (39)	73 (46)	58 (33)
III-IV	204 (61)	86 (54)	118 (67)
Echocardiographic			
Systolic LVF, *n* (%)			
Good (EF ≥ 50%)	326 (97)	155 (97)	171 (97)
Impaired (EF 40%–50%)	8 (2)	4 (3)	4 (2)
Moderately reduced (EF 30%–40%)	1 (0)	0	1 (0)
LAVI, mL/m^2^, median [IQR]	45 [37–55]	47 [37–59]	45 [37–54]
LADI, mm/m^2^, median [IQR]	21 [19–24]	22 [19–25]	21 [18–24]
Resting LVOT gradient, mmHg, median [IQR]	60 [38–81]	54 [31–76]	66 [46–87]
Provocative LVOT gradient, mmHg, mean (SD)^a^	85 (28)	85 (26)	87 (31)
Mitral regurgitation, *n* (%)			
Grade 1 + 2	231 (69)	112 (70)	119 (66)
Grade 3 + 4	103 (31)	47 (30)	56 (32)
Valvular SAM, *n* (%)	268 (80)	130 (82)	138 (78)
IVSd, mm, median [IQR]	21 [18–24]	21 [18–24]	21 [18–24]
TAPSE, mm, median [IQR]	20 [20–24]	21 [20–25]	21 [20–23]
Medication, *n* (%)			
ACE-inhibitors	59 (17)	32 (20)	27 (15)
Anticoagulation	154 (46)	73 (47)	81 (53)
Amiodarone	9 (2)	2 (1)	7 (4)
ARB	65 (19)	24 (15)	41 (23)
Beta-blockers	249 (74)	113 (71)	136 (77)
Calcium channel blockers	107 (32)	47 (30)	60 (34)
Disopyramide	16 (5)	8 (5)	8 (5)
Diuretics	86 (26)	37 (23)	49 (28)

Values are mean ± SD, median [IQR], or *n* (%).

Abbreviations: ACE, angiotensin-converting enzyme; ARB, angiotensin II receptor blocker; ASA, alcohol septal ablation; BMI, body mass index; EF, ejection fraction; ICD, implantable cardioverter defibrillator; IQR, interquartile range; IVSd, interventricular septal thickness in diastole; LADI, left atrial dimension index; LAVI, left atrial volume index; LBBB, left bundle branch block; LVF, left ventricular function; LVOT, left ventricular outflow tract; NYHA, New York Heart Association; RBBB, right bundle branch block; SAM, systolic anterior motion; SD, standard deviation; TAPSE, tricuspid annular plane systolic excursion.

^a^More than 50% missing and therefore calculated patients with outcome.

^b^Proportion of genotype-positive patients of genetically tested group.

**Table 2:** Intraoperative and 30-day clinical outcomes in patients operated on with surgical myectomy in The Netherlands stratified by high- or low-volume hospital

Characteristics	Overall (*n* = 335)	High-volume (*n* = 159)	Low-volume (*n* = 176)	*P-*value^a^
Intraoperative				
ACC time, min, median [IQR]	74 [46–126]	60 [41–99]	97 [53–142]	.008
ACC times, *n* (%)				.008
1	277 (83)	149 (94)	128 (73)	
2	50 (15)	9 (6)	41 (23)	
3	6 (2)	1 (1)	5 (3)	
4	1 (0)	0	1 (1)	
CPB time, min, median [IQR]	113 [75–142]	93 [66–139]	137 [83–201]	.008
Isolated surgical myectomy, *n* (%)	72 (22)	45 (28)	27 (15)	.008
Surgical myectomy with + 1 concomitant cardiac procedure, n (%)	181 (54)	78 (49)	103 (59)	.21
Surgical myectomy with 2 or more concomitant cardiac procedures, n (%)	82 (24)	36 (23)	46 (26)	.72
Concomitant surgery, *n* (%)				
Mitral valve surgery				
Replacement	52 (16)	16 (10)	36 (21)	.05
Repair	121 (36)	55 (35)	66 (38)	.78
CABG	45 (13)	26 (16)	19 (11)	.32
AVR	75 (22)	25 (16)	50 (28)	.05
TVR	6 (2)	2 (1)	4 (2)	.78
Aortic surgery	10 (3)	1 (1)	9 (5)	.07
Rhythm surgery	45 (13)	26 (16)	19 (11)	.31
Surgical myectomy, *n* (%)				NA
Transaortic	314 (94)	158 (99)	156 (89)	
Transseptal	12 (4)	0	12 (7)	
Waterston’s groove	7 (2)	0	7 (4)	
Transapical	0	0	0	
VSD^b^, *n* (%)	7 (2)	1 (1)	6 (3)	.25
Medication (at hospital discharge), *n* (%)				
ACE-inhibitors	83 (26)	37 (24)	46 (28)	.68
Anticoagulation	274 (82)	135 (87)	139 (84)	.78
Amiodarone	14 (4)	6 (4)	8 (5)	.89
ARB	40 (12)	14 (9)	26 (16)	.22
Beta-blockers	251 (75)	119 (76)	132 (80)	.70
Calcium channel blockers	57 (17)	17 (11)	40 (24)	.02
Disopyramide	3 (1)	1 (1)	2 (1)	NA
Diuretics	110 (33)	62 (40)	48 (29)	.18
Echocardiographic (first after surgical myectomy)				
Systolic LVF, *n* (%)				.34
Good (EF ≥ 50%)	286 (85)	134 (84)	152 (86)	
Impaired (EF 40%–50%)	37 (11)	22 (14)	15 (9)	
Moderately reduced (EF 30%–40%)	0	0	0	
LAVI, mL/m^2^, median [IQR]	44 [34–55]	45 [33–56]	46 [35–55]	.86
LADI, mm/m^2^, median [IQR]	22 [18–25]	22 [19–25]	21 [17–24]	.07
Resting LVOT gradient, mmHg, median [IQR]	9 [6–15]	8 [7–18]	11 [7–18]	.08
Provocative LVOT gradient, mmHg, mean (SD)^c^	17 (16)	10 (13)	33 (17)	.13
Residual LVOT gradient, *n* (%)				
Resting (≥ 30 mmHg)	24 (7)	8 (6)	16 (9)	.32
Provocative (≥ 50 mmHg)^d^	3 (1)	2 (1)	1 (1)	.94
Delta LVOT gradient, mmHg, mean (SD)	67 (29)	70 (26)	65 (29)	.25
Mitral regurgitation, *n* (%)				.72
Grade 1 + 2	304 (90)	145 (92)	159 (90)	
Grade 3 + 4	18 (5)	10 (6)	8 (5)	
Delta mitral regurgitation, *n* (%)				
Stable grade 1 + 2	217 (65)	107 (67)	110 (63)	.79
Stable grade 3 + 4	9 (3)	6 (4)	3 (2)	.52
Progression grade 1 + 2 to grade 3 + 4	9 (3)	4 (3)	5 (3)	NA
Regression grade 3 + 4 to grade 1 + 2	87 (26)	39 (25)	48 (27)	.68
Valvular SAM, *n* (%)	28 (8)	12 (8)	16 (9)	.78
IVSd, mm, median [IQR]	12 [10–14]	12 [10–13]	12 [10–13]	.58
TAPSE, mm, median [IQR]	17 [14–18]	16 [14–18]	16 [13–18]	.86
Postoperative (either at 30-day or during hospital admission), composite endpoint (11%; *n* = 38)				
Mortality, *n* (%)^b^	16 (5)	3 (2)	13 (7)	.07
Stroke, *n* (%)^b^	11 (3)	2 (1)	9 (5)	.23
Surgical reoperation, *n* (%)^b^				NA
Mitral valve replacement	2 (1)	0	2 (1)	
Mitral valve repair	3 (1)	2 (1)	1 (1)	
Myectomy	3 (1)	1 (1)	2 (1)	
New pacemaker, *n* (%)	34 (10)	15 (9)	19 (11)	.78
New ICD, *n* (%)	12 (4)	2 (1)	10 (6)	.12
Heart rhythm complications, *n* (%)	146 (44)	61 (38)	85 (48)	.22
LBBB, *n* (%)	227 (68)	103 (66)	124 (71)	.32
Mediastinitis, *n* (%)	4 (1)	2 (1)	2 (1)	NA
Bleeding, *n* (%)	32 (10)	10 (6)	22 (13)	.22
Hospital stay, days, median [IQR]	7 [6–12]	7 [6–11]	7 [6–12]	.78

Values are mean ± SD, median [IQR], or *n* (%).

Abbreviations: ACC, aortic cross-clamp; AVR, aortic valve replacement; CABG, coronary artery bypass grafting; CPB, cardiopulmonary bypass; EF, ejection fraction; ICD, implantable cardioverter defibrillator; IQR, interquartile range; IVSD, interventricular septal thickness in diastole; LADI, left atrial dimension index; LAVI, left atrial volume index; LBBB, left bundle branch block; LVF, left ventricular function; SAM, systolic anterior motion; TAPSE, tricuspid annular plane systolic excursion; TVR, tricuspid valve replacement; VSD, ventricular septal defect.

^a^False discovery rate correction was applied using the Benjamini-Hochberg method.

^b^Composite endpoint occurred in 11% of patients (*n* = 38).

^c^More than 50% missing and therefore calculated patients with outcome.

^d^Patients also experienced residual resting LVOT obstruction (≥ 30 mmHg).

### In-hospital echocardiographic outcomes

During a median follow-up of 8 [6–12] days, the mean resting LVOT gradient declined from 61 ± 30 mmHg preoperatively to 13 ± 12 mmHg postoperatively (mean difference: 47 mmHg [95% CI: 43 mmHg−50 mmHg]; *P* < .001). The surgical myectomy procedure was effective in 93% (*n* = 299) of patients postoperatively (95% CI: 89%–95%). A resting LVOT gradient ≥30 mmHg was present in 7% (*n* = 24) of patients postoperatively (95% CI: 5%–11%). A provocative LVOT gradient ≥30 mmHg, without a significant resting LVOT gradient, was postoperatively present in 0% (*n* = 0) of patients. Valvular SAM declined from 80% (*n* = 268) preoperatively to 8% (*n* = 28) postoperatively [difference in proportion: 71% (95% CI: 66%–77%)], and mitral regurgitation grade 3 or 4 improved from 31% (*n* = 103) preoperatively to 5% (*n* = 18) postoperatively [difference in proportion: 25% (95% CI: 20%–31%)].

### 30-day complication rates

The overall 30-day mortality rate was 5% [95% CI: 3%–8% (*n* = 16)]. Iatrogenic VSD rate was 2% [95% CI: 1%–4% (*n* = 7)], stroke rate was 3% [95% CI: 2%–6% (*n* = 11)], and overall surgical reoperation rate was 2% [95% CI: 1%–4%; surgical myectomy (0.9%; *n* = 3), mitral valve repair (0.9%; *n* = 3), mitral valve replacement (0.6%; *n* = 2)]. The composite endpoint occurred in 11% (*n* = 38) of patients (95% CI: 7%–14%). The sensitivity analyses for 30-day complication rates stratified for concomitant procedures are summarized in the Supplementary data online, *Table S8*. Stratification for surgical myectomy with two or more concomitant procedures is summarized in the Supplementary data online, *Table S9*. The mortality rate for isolated surgical myectomy was 0%. Firth’s logistic regression model demonstrated that having the female sex [OR 5.08 (95% CI: 2.17–11.87); *P* = .01], requiring two or more concomitant procedures [OR 6.54 (95% CI: 1.64–26.06); *P* = .05], and being operated on in a low-volume hospital [OR 3.58 (95% CI: 1.62–7.85); *P* = .01] were significantly associated with an increased risk of complication within 30 days after surgery. The results of multivariable analysis with Firth’s correction for 30-day complications are summarized in *Table 3*. Sensitivity analysis yielded a variance component intercept very close to zero, and therefore no clustering was observed after adjustment.

**Table 3:** Firth’s logistic regression for composite endpoint of 30-day complication rate

Factors	Multivariable model with firth correction		
	OR	95% CI	*P*-value^a^
Outcome: overall composite^b^ endpoint of 30-day complications			
Age	0.99	.96–1.02	.73
Female sex (vs. male sex)	5.08	2.17–11.87	.01
BMI ≥ 25 kg/m^2^ (vs. < 25)	0.81	.39–1.69	.79
Diabetes yes (vs. no)	3.07	1.17–8.09	.14
EuroSCORE II	0.92	.77–1.09	.60
Prior cardiac surgery yes (vs. no)	3.29	.60–17.97	.49
Prior ASA yes (vs. no)	1.42	.24–8.24	.82
Prior chronic lung disease yes (vs. no)	1.63	.57–4.63	.65
Prior neurologic dysfunction yes (vs. no)	0.16	.01–3.48	.54
Prior pacemaker yes (vs. no)	1.64	.23–11.48	.80
Prior RBBB yes (vs. no)	1.34	.05–.95	.82
Prior LBBB yes (vs. no)	0.72	.01–0.99	.80
Prior ACE-inhibitor (vs. no)	0.43	.14–1.32	.49
Prior amiodarone (vs. no)	1.51	.30–7.72	.80
Prior beta-blocker (vs. no)	0.80	.35–1.81	.80
Prior calcium channel blocker (vs. no)	0.99	.46–2.14	.99
Prior diuretics (vs. no)	1.71	.79–3.74	.49
NYHA functional class III-IV (vs. I-II)	0.60	.28–1.27	.49
At baseline LADI ≥ moderately abnormal (vs. ≤ mildly abnormal)	1.00	.39–2.55	.99
At baseline resting LVOT gradient	0.99	.98–1.01	.54
At baseline MV regurgitation grade 3 and 4 (vs. grade 1 and 2)	1.53	.73–3.21	.54
At baseline SAM yes (vs. no)	2.82	1.01–7.85	.27
Postoperative resting LVOT gradient in mmHg	0.99	.97–1.02	.85
Postoperative SAM yes (vs. no)	0.77	.18–3.33	.82
Surgical myectomy + 1 concomitant cardiac procedure (vs. isolated surgical myectomy)	2.99	.86–10.34	.36
Surgical myectomy + 2 or more concomitant cardiac procedure (vs. isolated surgical myectomy)	6.54	1.64–26.06	.05
Treatment surgical myectomy low-volume hospital (vs. surgical myectomy high-volume)	3.58	1.62–7.85	.01

Abbreviations: ASA, alcohol septum ablation; BMI, body mass index; CI, confidence interval; LADI, left atrial dimension index; LBBB, left bundle branch block; LVOT, left ventricular outflow tract; MV, mitral valve; OR, odds ratio; SAM, systolic anterior motion; VSD, ventricular septal defect.

^a^False discovery rate correction was applied using the Benjamini-Hochberg method.

^b^A composite of mortality, VSD, stroke, and surgical reoperation (myectomy, mitral valve repair, or mitral valve replacement).

### Longer-term outcomes

#### Overall survival

During a median follow-up of 4.0 [2.1–5.5] years, the overall mortality rate was 15% (*n* = 51) follow-up. Estimated overall survival rates for postoperative all-cause mortality at 1 and 5 years, were 93% (95% CI: 90%–96%), and 83% (95% CI: 78%–88%), respectively. Estimated survival rates using Kaplan-Meier method are documented in the Supplementary data online, *Figure S4*. Patient numbers declined substantially beyond 6 years of follow-up.

#### Morbidity

Between 30 days and end of follow-up, 10 patients (3%) underwent a second surgical procedure with a mean time to reintervention of 2 ± 1.45 years: eight redo mitral valve replacements, two surgical redo myectomy procedures, and zero redo mitral repairs.

At most recent follow-up, 162 (54%) of the patients were in NYHA functional class I, 110 (36%) in NYHA functional class II, 18 (6%) in NYHA functional class III, and 5 (2%) in NYHA functional class IV. NYHA functional class was unknown in 33 (10%) patients at the most recent follow-up.

### Hospital procedural volume

**Figure 1:**
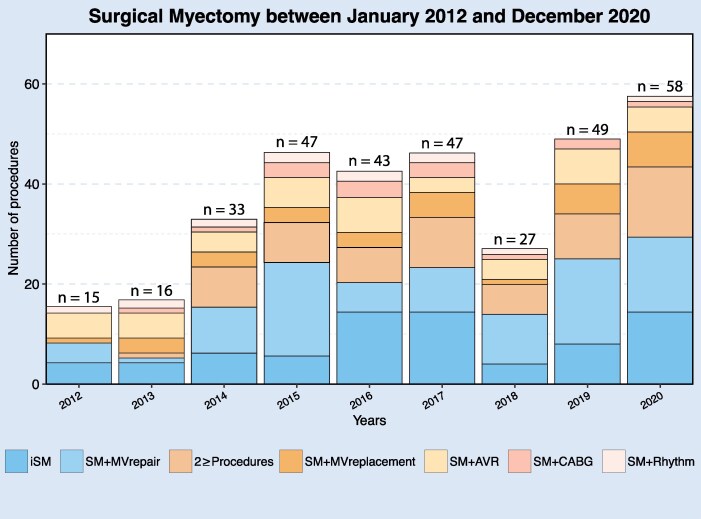
Incidence of surgical myectomy procedures per year from January 2012 to December 2020 in The Netherlands. The stacked bars represent the number of the specific surgical procedure (i.e. darker blue represents all patients with isolated surgical myectomy performed in that specific year). AVR, aortic valve replacement; CABG, coronary artery bypass grafting; iSM, isolated surgical myectomy; MV, mitral valve.

*Figure 1* depicts the annual number of surgical myectomy procedures stratified for concomitant procedures, which increased during the study period from 15 in 2012 to 58 in 2020. Among the 12 hospitals, two hospitals were classified as high volume, and 10 hospitals were classified as low volume. In high-volume hospitals, patients had a significantly lower all-cause mortality rate (2%) than patients in low-volume hospitals (7%; *P* < .001) at 30 days postoperatively. In high-volume hospitals, the incidence of iatrogenic VSD (1%) and stroke (1%) was not significantly different than in low-volume hospitals (3%, *P* = .12; and 5%, *P* = .10, respectively). In high-volume hospitals, surgical reoperation for a second surgical myectomy occurred in 1%, for mitral valve repair in 1%, and for mitral valve replacement in 0%, not different from the rates in low-volume hospitals (1%, 1%, and 1%, respectively).

## Discussion

This multicentre Dutch study describes the clinical outcomes of surgical myectomy in HOCM patients. In real-world clinical practice, surgical myectomy relieves LVOT obstruction in 93% of HOCM patients postoperatively. Second, the 30-day mortality for isolatedsurgical myectomy is 0%. Third, this is the first multicentre study to report a 30-day surgical reoperation rate (2%) following surgical myectomy and to provide detailed information on echocardiographic measurements. Fourth, this study identifies female sex, ≥ 2 concomitant procedures, and low-volume hospitals as significant factors associated with increased 30-day complication rates (Structured Graphical Abstract).

### Complications following surgical myectomy

The 30-day mortality rate (5%; 95% CI: 3%–8%) differs from analyses of previous Nationwide Inpatient databases conducted in the United States, where 30-day mortality was 3%.^13^ In-hospital mortality rates were 5%,^12^ 3%,^24^ and 4%.^11^ Heterogeneity in patient and treatment characteristics may explain the observed divergence in mortality rates. In the current study, the percentage of isolated surgical myectomy procedures performed was 22, compared with the cohort studies by Holst *et al*.^13^ (67%) and Altibi *et al*.^11^ (56%). This percentage of isolated surgical myectomy procedures in these multicentre studies can be attributed to these multicentre studies’ *a priori* exclusion of a significant portion of concomitant procedures, for example, surgical myectomy plus AVR. In the current study, patients operated on surgical myectomy plus AVR who were diagnosed with HOCM were not excluded due to diagnostic verification using electronic health records and echocardiographic images. The 2024 AHA/ACC guidelines for the management of HCM recommend achieving a mortality rate of <1% but is not clear whether this target solely applies to isolated surgical myectomy or also includes concomitant cardiac procedures.^25^ The 2023 ESC guidelines for the management of cardiomyopathy does not specify a target mortality rate.^1^ In the current study, the 30-day mortality rate after isolated surgical myectomy was 0% compared with the cohort studies by Holst *et al*.^13^ (< 1%), Wei *et al*.^26^ (2%), and Altibi *et al*.^11^ (2%). The stratified mortality rates are presented for the first time in a multicentre study for surgical myectomy plus mitral valve repair (1%), surgical myectomy plus AVR (2%), surgical myectomy plus CABG (0%), surgical myectomy plus rhythm surgery (0%), and surgical myectomy plus mitral valve replacement (19%). Single-centre studies presented different mortality rates for mitral valve repair (< 1%), surgical myectomy plus AVR (2%), and surgical myectomy plus CABG (2%).^27–30^ In the present study, surgical myectomy plus mitral valve replacement was performed in 27 patients. The relatively high 30-day mortality rate (19%, *n* = 5) in this group may be attributed to patient characteristics like comorbidity burden (endocarditis in *n* = 1), perioperative complications (iatrogenic VSD, *n* = 1), and postoperative complications (re-exploration for bleeding, *n* = 1; stroke, *n* = 2). Additionally, residual confounding by unmeasured factors is likely to have influenced the findings, including the extent of underlying mitral disease, operator volume and experience, variation in surgical technique, and institutional expertise. Nevertheless, this subgroup is relatively small; the rather high proportion is based on a limited number of events.^31–33^ Furthermore, at low-volume hospitals, more mitral valve replacement procedures were performed. Although no specific rationale was identified, this may reflect limited experience in managing SAM. Previous studies by Vassileva *et al*.^34^ and Faisaluddin *et al*.^14^ and have reported for surgical myectomy plus mitral valve replacement in-hospital mortality rates of 11% and 8%, respectively.

The in-hospital 30-day complications, such as stroke (3%) and complete heart block requiring pacemaker implantation (10%), were comparable to previous multicentre studies conducted in the United States by Kim *et al*.^12^ (2% and 10%, respectively) and Altibi *et al*.^11^ (2% and 10%, respectively). The implantation risk of a new permanent pacemaker (10% [*n* = 34]) can be explained by a pre-existence of right bundle branch block (RBBB). 42% of those with pre-existing RBBB patients (*n* = 9) underwent new permanent pacemaker implantation; this is in line with previous research that identified the need for new permanent pacemakers in 36% of patients with pre-existing RBBB.^27^ In patient who underwent ASA before surgery, the incidence of permanent pacemaker implantation was 23% (*n* = 4), which may be explained by RBBB pre-existence in 50% (*n* = 2) of these patients. Moreover, this represents a relatively small patient group, in which the proportion increases rapidly even with a limited number of events. In addition, the VSD rate (2%) was similar to that reported by Holst *et al*. (2%).^13^

### Echocardiographic outcomes

Nationwide studies have thus far omitted echocardiographic measurements after surgical myectomy in HOCM patients. In the present work, the overall resting LVOT gradient significantly declined from 61 ± 30 mmHg to 13 ± 12 mmHg postoperatively (mean difference, 47 mmHg; 95% CI: 43–50 mmHg; *P* < .001). In high-volume hospitals, the postoperative resting LVOT gradient of 11 ± 8 mmHg was significantly lower than in low-volume hospitals (15 ± 16 mmHg; *P* < .001). However, this might be explained by the lower preoperative resting LVOT gradient in high-volume hospitals (54 ± 29 mmHg) than in low-volume hospitals (66 ± 29 mmHg). High-volume single-centre studies have described lower or comparable resting LVOT gradients at hospital discharge: 2 ± 3 mmHg,^7^ 13 ± 19 mmHg,^10^ and 12 ± 9 mmHg.^9^ Low-volume single-centre studies have described comparable resting LVOT gradients at hospital discharge (19 ± 8 mmHg^35^ and 17 ± 8 mmHg.^36^)

### Hospital procedural volume

Low-volume hospital was associated with an increased risk of 30-day complications [OR 3.25 (95% CI: 1.41–8.25), *P* = .031], including mortality, VSD, stroke, and surgical reoperation after surgical myectomy. In high-volume hospitals, patients underwent isolated surgical myectomy in 28% compared with 15% in low-volume hospitals. Nevertheless, we adjusted for the categorised surgical procedures: (ⅰ) isolated surgical myectomy, (ⅱ) surgical myectomy with one concomitant procedure, and (ⅲ) surgical myectomy with two or more concomitant procedures. Previous literature on mortality described an increased in-hospital mortality risk for low-volume hospitals, for example, Holst *et al*.^13^ [OR 3.60 (95% CI: 2.03–6.38)], and Altibi *et al*.^11^ [OR 2.86 (95% CI: 1.70–4.80)]. There is no accepted definition of high- and low-volume hospitals for surgical myectomy. While Altibi *et al*.^11^ stratified the study cohort by tertiles into low-volume hospitals (< 8 surgical myectomy procedures/year), medium-volume hospitals (8–28 surgical myectomy procedures/year), and high-volume hospitals (> 28 surgical myectomy procedures/year), Holst *et al*.^13^ used other cut-offs and stratified the study cohort into low-volume hospitals (< 5 surgical myectomy procedures/year), medium-volume hospitals (5–10 surgical myectomy procedures/year), and high-volume hospitals (≥ 10 surgical myectomy procedures/year). Based on the present data and previous literature, a hospital procedural volume of 10 surgical procedures annually appears to be a reasonable threshold for more optimal results, with the understanding that selected lower-volume hospitals may also achieve excellent patient outcomes. We hope that these findings will encourage hospitals that perform surgical myectomy procedures to combine efforts to achieve more optimal results.

### Strengths and limitations

The main strength of the present study is that it used data from a multicentre registry and additionally considered detailed surgical, electrocardiographic, and echocardiographic variables. All echocardiographic measurements were examined by two experienced imaging cardiologists from a core laboratory. Moreover, the data have an exceptionally high degree of completeness. In addition, although referral bias due to the exclusion of non-participating centres (one academic and one non-academic) is a consideration—and the authors therefore cannot exclude the possibility that the findings of this study may not be generalisable to hospitals that did not participate—this study examines 12 out of 14 Dutch hospitals that performed surgical myectomy over a period of 9 years.

The study also has limitations. First, there was limited power to perform an analysis for individual complications (i.e. VSD, stroke, and surgical reoperation). This may have masked or diluted specific event rates. Although specific endpoints were carefully justified for inclusion in the composite outcome, the possibility of residual subjectivity in outcome attribution cannot be excluded [for example, iatrogenic VSD (related to surgical technique) and stroke (related to comorbidities)]. Second, there was limited power for stratified analyses by concomitant surgical myectomy procedures; therefore, surgical procedures were categorized into three subgroups to increase sample size. As a result, definitive conclusions for the presented subgroups could not be drawn, underscoring the need for further investigation in larger study populations to validate these findings. These findings should be considered hypothesis-generating for future studies. Third, the postoperative provocative LVOT gradient was missing in 80% of the patients, which may have led to missed or underestimated latent obstruction, potentially affecting the interpretation of the results. Fourth, at hospital discharge, echocardiographic data were collected, while at 30 days postoperatively, complication rates were evaluated. Fifth, there is a potential for selection bias given that 9% of patients did not provide informed consent (non-responders). Consequently, the overall cohort may not fully represent the entire population of patients with HOCM in the Netherlands. Sixth, although data collection was completed in 2020, the findings remain relevant to current clinical practice given the continued absence of large, comparable studies.

## Conclusion

This multicentre Dutch study describes clinical outcomes after surgical myectomy and demonstrates that this procedure effectively relieves LVOT obstruction in 93% of the patients. Isolated surgical septal myectomy is a safe procedure; however, surgical myectomy with ≥ 2 concomitant procedures, female sex, and low-volume hospitals are associated with elevated 30-day complication rates. Despite a significant increase in the number of surgical myectomies performed during the study period, the overall number of patients undergoing this procedure remains relatively small.

## Future perspective

The recent introduction of myosin inhibitors in the treatment of HOCM patients is expected to reduce the number of surgical myectomy procedures.^37^ In response to this change and to potentially achieve more optimal results, several novel strategies have been proposed: (ⅰ) the use of 3-dimensional printed models for improved preoperative planning and simulation training, (ⅱ) a minimally invasive beating-heart myectomy device, (ⅲ) thoracoscopic or robotic visualisation, (ⅳ) participation in surgical myectomy procedure at experienced hospitals, and (ⅴ) to increase the number of surgical myectomy procedures per hospital.^13,38–40^ However, further research is warranted to identify the impact of these possibilities on outcomes after surgical myectomy.

## Supplementary Material

ehaf560_Supplementary_Data

## Declarations

### Data Availability

The data supporting the findings of this study are available from the corresponding author upon reasonable request, subject to ethical and legal considerations.

### Pre-registered Clinical Trial Number

None supplied.

## Appendix

### SAM-Registry Study Investigators

J. Kluin, M. Palmen, R.G.H. Speekenbrink, F.R. Halfwerk, A. Yazdanbakhsh, L.A.F.M. van Garsse, R.C.A. Meijer, N.P.A. Zuithoff.

### Cardiothoracic Surgery Registration Committee of the Netherlands

Heart Registration: S. Bramer, R.A.F. De Lind Van Wijngaarden, B.M.J.A. Koene, J.A. Bekkers, G.J.F. Hoohenkerk, A.L.P. Markou, A. de Weger, P. Segers, D. Stecher, R.G.H. Speekenbrink, V.G. Hindori, W.W.L. Li, E.J. Daeter, M.M. Mokhles, Y.L. Douglas.
