# Supplementary material for: Surgical myectomy for hypertrophic cardiomyopathy: procedural volume and outcomes
Source: Eur Heart J. 2025 Aug 29;47(20):2481–93. doi: 10.1093/eurheartj/ehaf560 (PMC13191829; doi:10.1093/eurheartj/ehaf560)
Supplement: ehaf560_Supplementary_Data [file ehaf560_supplementary_data.pdf]

Supplementary material

Hospital Volume and Real-World Outcomes After Surgical Myectomy:

Data of the Netherlands Heart Registration

Corresponding Author:

Niels. P. van der Kaaij

Erasmus University Medical Centre, Department of Cardiothoracic Surgery

Dr. Molenwaterplein 40, 3015 GD, Rotterdam, Netherlands

Telephone: +31 6 8655 3194

Email: n.vanderkaaij@erasmusmc.nl

Data S1.

Supplemental Methods

Database:
We queried the Netherlands Heart Registration (NHR) for the periprocedural variable "myectomy" performed between January 1, 2012, and December 31, 2020. All fifteen Dutch cardiac surgical hospitals register their interventions in the NHR quality registry database. Multiple quality checks are performed on the data during the data collection and cardiac hospitals are audited annually.[1,2] In this study, a total of twelve hospitals participated in the study. While one hospital did not perform any surgical myectomy procedure, the other two hospitals did consent to participate in this study. The present study was deemed exempt by the Institutional Review Boards of all 12 participating hospitals and informed consent was required as the study used additional patient data (**supplementary Table S3**).

Routinely collected NHR variables
The following data from the national database of the NHR were used for this study. These data were manually reviewed from the electronic health records to check, complete, and adjust the NHR data where needed. The sets of variables and their definitions are available on the NHR website (https://nhr.nl/handboeken/#handboeken).

<u>Data on Patient and Hospital Characteristics:</u>
Data on patient demographics (age, gender, length, and height), and comorbidities (diabetes mellitus, chronic lung disease, dialysis, extracardiac chronic arterial pathology, left ventricular function, neurologic dysfunction, active endocarditis, recent myocardial infarction, pulmonary artery pressure, prior cerebrovascular accident, mobility, unstable angina, preoperative state, prior cardiac surgery, prior percutaneous coronary intervention, prior aortic surgery, EuroSCORE I, EuroSCORE II, New York Heart Association (NYHA) functional class, procedural urgency, atrial fibrillation, permanent pacemaker (PPM), and implantable cardioverter defibrillator (ICD)) were extracted.

<u>Data on the surgical procedure:</u>
Data on concomitant surgery (coronary artery bypass grafting, mitral valve replacement, mitral valve repair, mitral valve implant, aortic valve (AV) replacement, AV repair, tricuspid valve surgery, pulmonary artery surgery, aortic surgery, atrial septal closure, ventricular septal closure, cardiac assist device, heart rupture closure, ICD implantation, PM implantation, pericardiectomy, heart aneurism, rhythm surgery), extracorporeal circulation time, and aortic cross-clamp (ACC) time were extracted.

<u>Data on the post-surgical procedure and follow-up:</u>
The follow-up data was collected until the end of the follow-up. Data was collected in twelve different hospitals consecutively. The submission of data from the first hospital occurred in December 2022, and the submission of data from the last hospital occurred in June 2023.
Data on in-hospital mortality, mortality until and of follow-up, admission days, pneumonia, urinary tract infection, readmission to intensive care unit, CVA with permanent damage, CVA without permanent damage, renal failure, vascular complication, heart rhythm complications, rethoracotomy, deep sternal wound infection, and readmission intensive care unit (ICU) were extracted

Additional NHR variables
For this study, the following data were collected in addition to NHR variables. The data is collected for the national database of the NHR, and their definitions are available on the NHR website (https://nhr.nl/handboeken/#handboeken).

<u>Data on patient characteristics (pre- and postoperative at hospital discharge; *only collected pre-operatively)</u>

Data on heart rate, AV Block (first-degree AV block, second-degree AV block, Mobitz I, Mobitz II, and third-degree AV block), left bundle branch block, right bundle branch block, blood pressure, medication (beta-blockers, calcium antagonist, disopyramide, diuretics, anticoagulant, amiodaron, ACE-inhibitor, angiotensin II receptor blocker), prior alcohol septal ablation, and pathogenetic DNA variant* were extracted.*

Data on echocardiographic measurements (pre- and postoperatively at hospital discharge)
Data on resting left ventricular outflow tract (LVOT) gradient, provocative LVOT gradient, provocative maneuver (valsalva, exercise, or dobutamine), mitral regurgitation, aortic regurgitation, aortic valve stenosis, planimetry, valvular systolic anterior motion (SAM), interventricular septal thickness in diastole, tricuspid annular plane systolic excursion, left atrial dimension and left ventricular ejection fraction were extracted.

Data on surgical procedure:
Data on the amount of re-ACC, indication re-ACC, approach (transaortic, transseptal, Waterston's groove, transapical), mitral valve repair techniques (edge-to-edge [Alfieri stitch], anterior mitral valve leaflet extension, secondary chordal cutting, papillary muscle mobilization, annuloplasty ring, neo chordae, plication posterior mitral valve leaflet), indication mitral valve repair (intrinsic (papillary muscle/ chordae rupture, prolapse, stenosis, annulus dilatation, myxomatous, endocarditis), non-intrinsic (SAM mediated MR, SAM, preventive)), surgeon, and amount of myectomy procedures performed by the surgeon were extracted.

Data on post-surgical procedure and follow-up:
The follow-up data was collected with a follow-up period of 30 days. Data was collected in 12 hospitals. The submission of data from the first hospital occurred in December 2022, and the submission of data from the last hospital occurred in June 2023.
Data on ICU admission, 30-day new permanent pacemaker implantation, NYHA functional class, surgical reoperation (surgical myectomy, mitral valve repair and/or mitral valve replacement), and other interventions (ICD, PM, radiofrequency ablation, alcohol septal ablation) were extracted.

Supplemental Tables

Supplementary Table S1. Members of the Cardiothoracic Surgery Registration Committee of the Netherlands Heart Registration.

Members name			Hospital
S.		Bramer	Amphia Ziekenhuis
R.A.F.		De Lind van Wijngaarde	Amsterdam UMC, locatie AMC
B.M.J.A.		Koene	Catharina Ziekenhuis
J.A.		Bekkers	Erasmus Medisch Centrum
G.J.F.		Hoohenkerk	HagaZiekenhuis
A.L.P.		Markou	Isala
A.	de	Weger	Leids Universitair Medisch Centrum
P.		Segers	Maastricht UMC+
D.		Stecher	Medisch Centrum Leeuwarden
R.G.H.		Speekenbrink	Medisch Spectrum Twente
V.G.		Hindori	OLVG
W.W.L.		Li	Radboudumc
E. J.		Daeter	St. Antonius Ziekenhuis
M.M.		Mokhles	Universitair Medisch Centrum Utrecht
Y.L.		Douglas	Universitair Medisch Centrum Groningen

Supplementary Table S2. Members of the Study Group (SAM-Registry Study Investigators).

Name			Hospital
J.		Kluin	Erasmus MC
M.		Palmen	Leids Universitair Medisch Centrum, Amsterdam Universitair Medisch Centrum
R.G.H.		Speekenbrink	Medisch Spectrum Twente

F.R.		Halfwerk	Medisch Spectrum Twente
A.		Yazdanbakhsh	OLVG
L.A.		Garsse	Radboudumc
R.C.A.		Meijer	Universitair Medisch Centrum Utrecht
P.		Zuithoff	Universitair Medisch Centrum Utrecht

Supplementary Table S3. Approval from all Institutional Review Boards.

Institutional Review Board	Number
Catharina hospital	nWMO-2022.043
Haga hospital	G21.159
Isala hospital Zwolle	210511
Leiden University Medical Centre	2022-052
Maastricht University Medical Centre	2021-2915
Medisch Spectrum Twente	KH21-34
OLVG hospital	WO 21.114
St. Antonius Nieuwegein	R&D/Z21.063
University Medical Centre Nijmegen	2021-13076
University Medical Centre Utrecht	WO 21.114
University Medical Centre Groningen & Amsterdam University Medical Centre	Waived the need for approval because of prior approval at UMCU (WO 21.114) and acknowledged that decision.

Supplementary Table S4. Missing values of preoperative patient characteristics of all included surgical myectomy procedures

	Total (n = 335)	High volume (n = 159)	Low volume (n = 176)
**Age**	**0 (0%)**	0 (0%)	0 (0%)
**Sex**	**0 (0%)**	0 (0%)	0 (0%)
**BMI in kg/m^2**	**0 (0%)**	0 (0%)	0 (0%)
**Diabetes**	**0 (0%)**	0 (0%)	0 (0%)
**Creatinine in umol/l**	**0 (0%)**	0 (0%)	0 (0%)
**EuroSCORE II**	**0 (0%)**	0 (0%)	0 (0%)
**Prior cardiac surgery**	**0 (0%)**	0 (0%)	0 (0%)
**Prior ASA**	**1 (0 %)**	0 (0%)	0 (0%)
**Chronic lung disease**	**0 (0%)**	0 (0%)	0 (0%)
**Extracardiac arteriopathy**	**0 (0%)**	0 (0%)	0 (0%)

**Neurologic dysfunction**	**0 (0%)**	0 (0%)	0 (0%)
**Atrial fibrillation history**	**6 (2%)**	0 (%)	6 (3%)
**ICD history**	**0 (0%)**	0 (0%)	0 (0%)
**Pacemaker history**	**1 (0%)**	0 (%)	1 (0%)
**LBBB**	**1 (0%)**	0 (%)	1(0%)
**RBBB**	**1 (0%)**	0 (%)	1 (0%)
**Emergency operation**	**0 (0%)**	0 (0%)	0 (0%)
**Endocarditis**	**0 (0%)**	0 (0%)	0 (0%)
**Pathogenetic DNA variants**	**183 (55%)**	104 (65 %)	79 (45%)
**Family history of HCM (first degree)**	**7 (2%)**	0 (0%)	7 (4%)
**NYHA functional class**	**0 (0%)**	0 (0%)	0 (0%)
**Systolic LVF**	**1 (0%)**	0 (0%)	0 (0%)
**LAVI**	**47 (14%)**	40 (25%)	7 (4%)
**LADI**	**20 (6%)**	19 (12%)	1 (1%)
**Resting LVOT gradient**	**0 (%)**	0 (0%)	0 (0%)
**Provocative LVOT gradient**	**167 (50%)**	61 (37%)	106 (60%)
**Mitral regurgitation**	**1 (0%)**	0 (0%)	1 (0%)
**Valvular SAM**	**0 (0%)**	0 (0%)	0 (0%)
**IVSD**	**0 (0%)**	0 (0%)	0 (0%)
**TAPSE**	**3 (1%)**	2 (1%)	1 (0%)
**ACE-inhibitors**	**0 (0%)**	0 (0%)	0 (0%)
**Anticoagulantia**	**0 (0%)**	0 (0%)	0 (0%)
**ARB**	**0 (0%)**	0 (0%)	0 (0%)
**B-blockers**	**0 (0%)**	0 (0%)	0 (0%)
**Calcium channel blockers**	**0 (0%)**	0 (0%)	0 (0%)
**Disopyramide**	**0 (0%)**	0 (0%)	0 (0%)
**Diuretics**	**0 (0%)**	0 (0%)	0 (0%)

Abbreviations: ACE, angiotensin-converting enzyme; ARB, angiotensin II receptor blocker; ASA, alcohol septal ablation; BMI, body mass index; ICD, implantable cardioverter defibrillator IVSD, interventricular septal thickness in diastole; LADI, left atrial dimension index; LAVI, left atrial volume index; LBBB, left bundle branch block; LVF, left ventricular function; LVOT, left ventricular outflow tract; NYHA, New York Heart Association; RBBB, right bundle branch block; SAM, systolic anterior motion; TAPSE, tricuspid annular plane systolic excursion.

Supplementary Table S5. Missing values of intra-operative, and postoperative variables of all included surgical myectomy procedures.

	Total (n = 335)	High volume (n = 159)	Low volume (n = 176)
**VSD**	**0 (0%)**	0 (0%)	0 (0%)
**ACC time in minutes**	**26 (8%)**	18 (11%)	8 (5%)
**ACC times**	**0 (0%)**	0 (0%)	0 (0%)
**CPB time in minutes**	**26 (8%)**	18 (11%)	8 (5%)
**Concomitant surgery**	**0 (0%)**	0 (0%)	0 (0%)
**Surgical myectomy**	**0 (0%)**	0 (0%)	0 (0%)
**Mortality**	**0 (0%)**	0 (0%)	0 (0%)
**New pacemaker**	**10 (3%)**	0 (0%)	10 (6%)
**New ICD**	**4 (1%)**	0 (0%)	0 (0%)
**Stroke**	**1 (0%)**	0 (0%)	0 (0%)
**Heart rhytm complications**	**0 (0%)**	0 (0%)	0 (0%)
**LBBB postoperative**	**4 (1%)**	3 (2%)	1 (0%)
**Surgical reoperation**	**4 (1%)**	3 (2%)	1 (0%)
**Mediastinitis**	**15 (4%)**	0 (0%)	15 (8%)
**Bleeding**	**0 (0%)**	0 (0%)	0 (0%)
**Hospital stay in days**	**0 (0%)**	0 (0%)	0 (0%)
**Systolic LVF**	**12 (4%)**	3 (2%)	0 (0%)
**LAVI**	**33 (10%)**	15 (9%)	18 (10%)
**LADI**	**22 (6%)**	10 (6%)	12 (7%)
**Resting LVOT gradient**	**13 (4%)**	3 (2%)	10 (6%)
**Provocative LVOT gradient**	**276 (80%)**	109 (69%)	167 (95%)
**Mitral regurgitation**	**12 (4%)**	3 (2%)	9 (5%)
**Valvular SAM**	**13 (4%)**	4 (3%)	9 (5%)
**IVSd**	**22 (7%)**	10 (6%)	12 (7%)
**TAPSE**	**14 (4%)**	3 (2%)	11 (6%)

Abbreviations: ACC, aortic cross clamp; CPB, cardiopulmonary bypass; ICD, implantable cardioverter defibrillator; IVSD, inter ventricular septal thickness in diastole; LADI, left atrial dimension index; LAVI, left atrial volume index; LBBB, left bundle branch block; LVF, left ventricular function; SAM, systolic anterior motion; TAPSE, tricuspid annular plane systolic excursion; VSD, ventricular septal defect.

Table S6. Patient characteristics of HOCM patients operated on with surgical myectomy with a history of alcohol septal ablation in the Netherlands stratified by high- or low-volume hospital.

Characteristics		Overall	ASA subgroup
		(n = 335)	(n = 13)
Age in years (mean, [SD])		*65 [56–70]*	*62 [50–67]*
Sex (male, n [%])		*179 (53)*	*9 (69)*
BMI in kg/m² (mean, [SD])		*27 [24–31]*	*26 [24–29]*
Diabetes (n, [%])		*56 (17)*	*0*
Creatinine in µmol/l (median, [IQR])		*81 [71–96]*	*86 [75–90]*
EuroSCORE II (median, [IQR])		*2 [1–3]*	*1 [1–4]*
Prior cardiac surgery (n, [%])		*16 (5)*	*2 (15)*
Prior ASA (n, [%])		*13 (4)*	*13 (100)*
Chronic lung disease (n, [%])		*40 (12)*	*1 (8)*
Extracardiac arteriopathy (n, [%])		*15 (4)*	*1 (8)*
Neurologic dysfunction (n, [%])		*7 (2)*	*0*
Atrial fibrillation history (n, [%])		*65 (19)*	*2 (15)*
ICD history (n, [%])		*22 (7)*	*0*
Pacemaker history (n, [%])		*9 (3)*	*0*
LBBB (n, [%])		*33 (10)*	*0*
RBBB (n, [%])		*24 (7)*	*4 (31)*
Emergency operation (n, [%])		*5 (2)*	*0*
Endocarditis (n, [%])		*3 (9)*	*0*
*Pathogenetic DNA variants * & **(n, [%])*		*87 (58)*	*4 (57)*
Family history of HCM (first-degree relative)		*55 (16)*	*4 (31)*
NYHA functional class (n, [%])			
	I & II	*131 (39)*	*5 (38)*
	III & IV	*204 (61)*	*8 (62)*
Echocardiographic			
Systolic LVF (n, [%])			
	Good (EF ≥ 50%)	*326 (97)*	*13 (100)*
	Impaired (EF 40%–50%)	*8 (2)*	*0*
	Moderately reduced (EF 30%–40%)	*1 (0)*	*0*
LAVI in mL/m² (mean, [SD])		*45 [37–55]*	*42 [39–43]*
LADI in mm/m² (mean, [SD]))		*21 [19–24]*	*23 [21–25]*

Resting LVOT gradient (mean, [SD])		*60 [38–81]*	*60 [51–66]*
*Provocative LVOT gradient (mean, [SD])***		*85 (28)*	*83 (13)*
Mitral regurgitation (n, [%])			
	Grade 1 + 2	*231 (69)*	*8 (62)*
	Grade 3 + 4	*103 (31)*	*5 (38)*
Valvular SAM (n (%))		*268 (80)*	*12 (92)*
IVSd in mm (mean, [SD])		*21 [18–24]*	*19 [18–21]*
TAPSE in mm (mean, [SD])		*20 [20–24]*	*22 [20–24]*
Medication (n, [%])			
	ACE-inhibitors	*59 (17)*	*4 (31)*
	Anticoagulation	*154 (46)*	*7 (54)*
	Amiodaron	*9 (2)*	*0*
	ARB	*65 (19)*	*1 (8)*
	B-blockers	*249 (74)*	*10 (77)*
	Calcium channel blockers	*107 (32)*	*4 (31)*
	Disopyramide	*16 (5)*	*1 (8)*
	Diuretics	*86 (26)*	*1 (8)*

*Values are mean ± standard deviation (SD), median [IQR], or n (%). Abbreviations: ACE, angiotensin-converting enzyme; ARB, angiotensin II receptor blocker; ASA, alcohol septal ablation; BMI, body mass index; ICD, implantable cardioverter defibrillator; IQR, interquartile range; IVSD, interventricular septal thickness in diastole; LADI, left atrial dimension index (mm/m^2); LAVI, left atrial volume index (mL/m2); LBBB, left bundle branch block; LVF, left ventricular function; LVOT, left ventricular outflow tract; NYHA, New York Heart Association; RBBB, right bundle branch block; SAM, systolic anterior motion; SD, standard deviation; TAPSE, tricuspid annular plane systolic excursion. *more than 50% missing and therefore calculated patients with outcome, ** proportion of genotype-positive patients of genetically tested group.*

Table S7. Intraoperative and 30-day clinical outcomes in patients operated on with surgical myectomy with a history of alcohol septal ablation in the Netherlands stratified by high- or low-volume hospital.

Characteristics			Overall	ASA subgroup
			(n = 335)	(n = 13)
Intraoperative				
	ACC time in minutes (median, [IQR])		*74 [46–126]*	*63 [61–94]*
	CPB time in minutes (median, [IQR])		*113 [75–142]*	*103 [94–170]*
	Concomitant surgery (n, [%])			
		Isolated myectomy	*72 (22)*	*2 (15)*

Mitral valve surgery		
Replacement	*52 (16)*	*1 (8)*
Repair	*121 (36)*	*5 (38)*
CABG	*45 (13)*	*0*
AVR	*75 (22)*	*3 (23)*
Rhythm surgery	*45 (13)*	*3 (23)*
*VSD (n, [%])**	*7 (2)*	*0*
Echocardiographic (first after surgical myectomy)		
Systolic LVF (n, [%])		
Good (EF ≥ 50%)	*286 (85)*	*134 (84)*
Impaired (EF 40%–50%)	*37 (11)*	*22 (14)*
Moderately reduced (EF 30%–40%)	*0*	*0*
LAVI in mL/m^2 (median, [IQR])	*44 [34–55]*	*41 [37–47]*
LADI in mm/m^2 (median, [IQR])	*22 [18–25]*	*22 [19–25]*
Resting LVOT gradient (median, [IQR])	*9 [6–15]*	*8 [7–18]*
*Provocative LVOT gradient ((mean, [SD])***	*17 (16)*	*10 (13)*
Residual LVOT gradient		
Resting (≥ 30 mmHg) (n, [%])	*24 (7)*	*8 (6)*
*Provocative (≥ 50 mmHg) (n, [%]) ****	*3 (1)*	*2 (1)*
Mitral regurgitation (n, [%])		
Grade 1 + 2	*304 (90)*	*145 (92)*
Grade 3 + 4	*18 (5)*	*10 (6)*
Valvular SAM (n [%])	*28 (8)*	*12 (8)*
IVSd in mm (median, [IQR])	*12 (10–14)*	*12 (10–14)*
TAPSE in mm (median, [IQR])	*17 [14–18]*	*17 [14–19]*
Postoperative (either at 30-day or during hospital admission), composite endpoint (11%,; n = 38)		
*Mortality (n, [%])**	*16 (5)*	*0*
*Stroke (n, [%])**	*11 (3)*	*2 (1)*
*Surgical reoperation (n, [%])**		
Mitral valve replacement	*2 (1)*	*0*
Mitral valve repair	*3 (1)*	*0*
Myectomy	*3 (1)*	*0*
New pacemaker (n, [%])	*34 (10)*	*3 (23)*
New ICD (n, [%])	*12 (4)*	*1 (8)*
Heart rhythm complications (n, [%])	*146 (44)*	*61 (38)*
LBBB (n, [%])	*227 (68)*	*103 (66)*

	Mediastinitis (n, [%])	*4 (1)*	*2 (1)*
	Bleeding (n, [%])	*32 (10)*	*10 (6)*
	Hospital stay in days (median, [IQR])	*7 [6–12]*	*7 [6–11]*

*Values are mean ± standard deviation (SD), median [IQR] or n (%). Abbreviations: ACC, aortic cross-clamp; AVR, aortic valve replacement; CABG, coronary artery bypass grafting; CPB, cardiopulmonary bypass; ICD, implantable cardioverter defibrillator; IQR, interquartile range; IVSd, interventricular septal thickness in diastole; LADI, left atrial dimension index (mm/m^2); LAVI, left atrial volume index (mL/m^2); LBBB, left bundle branch block; LVF, left ventricular function; SAM, systolic anterior motion; TAPSE, tricuspid annular plane systolic excursion; TVR, tricuspid valve replacement; VSD, ventricular septal defect. *Composite endpoint occurred in 11% of patients (n = 38) , **more than 50% missing and therefore calculated patients with outcome, *** patients experienced also residual resting LVOT obstruction (≥ 30 mmHg).*

Supplementary Table S8. Sensitivity analyses for clinical outcomes stratified for concomitant surgical procedures.

Characteristics	Overall	Isolated SM	SM + MV Repair	SM + MV replacement	SM + CABG	SM + AVR	SM + rhythm surgery	SM + ≥ 2 procedures
	(n = 335)	(n = 72; 22%)	(n = 82; 25%)	(n = 27; 8%)	(n = 14; 4%)	(n = 41; 12%)	(n = 15; 5%)	(n = 8; 25%)
Intraoperative								
VSD (n, [%])	*7 (2)*	*1 (1)*	*2 (2)*	*1 (3)*	*0*	*0*	*1 (7)*	*2 (3)*
ACC time in minutes (median, [IQR])	*74 [46–126]*	*40 [28–58]*	*85 [57–121]*	*140 [112–210]*	*69 [34–95]*	*68 [51–93]*	*77 [58–109]*	*131 [78–197]*
ACC times (n, [%])								
1	*277 (83)*	*62 (86)*	*80 (87)*	*22 (69)*	*15 (100)*	*44 (96)*	*12 (80)*	*42 (69)*
2	*50 (15)*	*10 (14)*	*12 (13)*	*9 (28)*	*0*	*2 (4)*	*2 (13)*	*15 (25)*
3	*6 (2)*	*0*	*1 (3)*	*1 (3)*	*0*	*0*	*0*	*4 (7)*
4	*1 (0)*	*0 (0)*	*0*	*0*	*0*	*0*	*1 (7)*	*0*
CPB time in minutes (median, [IQR])	*113 [75–189]*	*63 [52–94]*	*118 [81–165]*	*209 [172–288]*	*100 [73–132]*	*96 [71–124]*	*106 [79–148]*	*200 [120–254]*
Echocardiographic (first after surgical myectomy)								
Systolic LVF (n, [%])								
Good (EF ≥ 50%)	*286 (85)*	*66 (92)*	*81 (88)*	*25 (78)*	*15 (100)*	*42 (91)*	*11 (73)*	*47 (75)*
Impaired (EF 40%–50%)	*37 (11)*	*6 (8)*	*11 (12)*	*2 (6)*	*0*	*2 (4)*	*4 (27)*	*12 (19)*
Moderately reduced (EF 30%–40%)	*0*	*0*	*0*	*0*	*0*	*0*	*0*	*0*
LAVI in mL/m² (median, [IQR])	*44 [34–55]*	*44 [33–59]*	*41 [34–50]*	*50 [41–55]*	*37 [25–61]*	*52 [42–57]*	*47 [41–49]*	*43 [33–54]*
LADI in mm/m² (median, [IQR])	*22 [18–25]*	*21 [18–23]*	*19 [17–23]*	*21 [20–22]*	*23 [18–26]*	*21 [20–23]*	*22 [20–27]*	*24 [20–26]*
Resting LVOT gradient in mmHg (median, [IQR])	*9 [6–15]*	*9 [6–15]*	*13 [7–18]*	*7 [6–10]*	*7 [5–9]*	*11 [6–16]*	*10 [6–17]*	*9 [6–12]*
*Provocative LVOT gradient in mmHg ((mean, [SD])**	*17 (16)*	*18 (18)*	*18 (17)*	*22 (22)*	*5 (2)*	*12 (12)*	*22 (25)*	*9 (4)*
Mitral regurgitation (n, [%])								
Grade 1 + 2	*310 (93)*	*69 (96)*	*83 (90)*	*22 (82)*	*11 (73)*	*42 (91)*	*15 (100)*	*54 (86)*
Grade 3 + 4	*19 (6)*	*3 (4)*	*5 (5)*	*0*	*4 (27)*	*2 (4)*	*0*	*3 (5)*
Valvular SAM (n [%])	*28 (8)*	*9 (13)*	*9 (9)*	*0*	*0*	*3 (7)*	*2 (13)*	*5 (8)*
IVSd in mm (median, [IQR])	*12 [10–14]*	*12 [9–13]*	*12 [10–14]*	*12 [10–13]*	*11 [9–13]*	*11 [10–13]*	*10 [9–13]*	*11 [10–13]*
TAPSE in mm (median, [IQR])	*17 [14–18]*	*17 [14–18]*	*17 [14–20]*	*17 [16–18]*	*13 [13–15]*	*16 [15–17]*	*16 [14–17]*	*15 [12–18]*
Postoperative (either at 30-day or during hospital admission)								
Mortality (n, [%])	*16 (5)*	*0*	*1 (1)*	*5 (19)*	*0*	*1 (2)*	*0*	*6 (10)*
New Pacemaker (n, [%])	*34 (10)*	*8 (12)*	*7 (8)*	*1 (3)*	*1 (7)*	*6 (14)*	*1 (7)*	*10 (17)*
New ICD (n, [%])	*24 (7)*	*5 (8)*	*1 (4)*	*1 (3)*	*1 (7)*	*3 (2)*	*2 (13)*	*6 (7)*
Stroke (n, [%])	*11 (3)*	*0*	*4 (4)*	*4 (15)*	*1 (7)*	*1 (2)*	*0*	*1 (2)*
Heart rhythm complications (n, [%])	*146 (44)*	*30 (42)*	*43 (47)*	*15 (47)*	*8 (54)*	*17 (37)*	*6 (40)*	*27 (43)*
LBBB (n, [%])	*227 (68)*	*59 (82)*	*69 (75)*	*22 (74)*	*9 (60)*	*28 (61)*	*12 (80)*	*33 (54)*
Mitral valve surgery	*5 (1)*	*1 (1)*	*3 (3)*	*0*	*0*	*0*	*0*	*1 (2)*
Myectomy	*3 (1)*	*1 (1)*	*1 (1)*	*0*	*0*	*0*	*0*	*1 (2)*
Mediastinitis	*4 (1)*	*0 (0)*	*1 (1)*	*0*	*1 (7)*	*0*	*1 (7)*	*1 (2)*
Bleeding	*32 (10)*	*4 (6)*	*6 (7)*	*4 (15)*	*0*	*5 (11)*	*4 (27)*	*9 (15)*

Values are mean ± standard deviation (SD), median [IQR] or n (%). Abbreviations: ACC, aortic cross clamp; AVR, aortic valve replacement; CABG, coronary artery bypass grafting; CPB, cardiopulmonary bypass; ICD, implantable cardioverter defibrillator; IQR, interquartile range; IVSD, inter ventricular septal thickness in diastole; LADI, left atrial dimension index; LAVI, left atrial volume index; LBBB, left bundle branch block; LVF, left ventricular function; SAM, systolic anterior motion; TAPSE, tricuspid annular plane systolic excursion; TVR, tricuspid valve replacement; VSD, ventricular septal defect.

Table S9. Intraoperative two or more surgical procedures concomitant to surgical myectomy in patients with hypertrophic obstructive cardiomyopathy

Surgical procedure		Overall
		(n = 82)
Two concomitant surgical procedures (n, [%])		
	MV replacement + AVR	11 (13)
	MV replacement + rhythm surgery	6 (6)
	MV replacement + aortic surgery	1 (1)
	MV replacement + CABG	3 (2)
	CABG + MV repair	10 (12)
	CABG + AVR	9 (11)
	CABG + rhythm surgery	1 (1)
	MV repair + rhythm surgery	10 (12)
	MV repair + AVR	5 (6)
	MV repair + aortic surgery	1 (1)
	MV repair + MV replacement	2 (2)
	AVR + rhythm surgery	5 (6)
	AVR + aortic surgery	2 (2)
Three concomitant surgical procedures (n, [%])		
	MV replacement + AVR + rhythm surgery	1 (1)
	CABG + MV repair + rhythm surgery	4 (5)
	CABG + AVR + rhythm surgery	1 (1)
	CABG + MV repair + AVR	1 (1)
	CABG + MVR + AVR	1 (1)
	MV repair + aortic surgery + AVR	2 (2)
	MV repair + rhythm surgery + TVR	2 (2)
	AVR + TVR + aortic surgery	1 (1)
Four concomitant surgical procedures (n, [%])		
	MV repair + aortic surgery + AVR + TVR	2 (2)
	CABG + MV replacement + AVR + rhythm surgery	1 (1)

Values are presented as number (%). Abbreviations: AVR, aortic valve replacement; CABG, coronary artery bypass grafting; MV, mitral valve; TVR, tricuspid valve replacement.

Table 10. Patient characteristics of HOCM patients operated on with surgical myectomy in the Netherlands stratified by mitral valve surgery.

Characteristics	Overall	Isolated surgical myectomy	Surgical myectomy with E2E	Surgical myectomy with AMLE	Surgical myectomy with MV replacement
	(n = 335)	(n = 72)	(n = 30)	(n = 36)	(n = 27)
Age in years (median, [IQR])	*65 [56–70]*	*63 [54–61]*	*61 [50–68]*	*62 [51–68]*	*66 [61–60]*
Sex (male, n [%])	*179 (53)*	*42 (58)*	*16 (53)*	*19 (53)*	*12 (44)*
BMI in kg/m² (median, [IQR])	*27 [24–31]*	*30 [25–32]*	*30 [25–32]*	*26 [23–29]*	*27 [25–29]*
Diabetes (n, [%])	*56 (17)*	*4 (6)*	*6 (20)*	*1 (3)*	*2 (7)*
Creatinine in µmol/l (median, [IQR])	*81 [71–96]*	*82 [69–92]*	*82 [72–99]*	*77 [72–87]*	*79 [69–107]*
EuroSCORE II (median, [IQR])	*2 [1–3]*	*1 [1–2]*	*3 [1–3]*	*2 [1–2]*	*2 [2–4]*
Prior cardiac surgery (n, [%])	*16 (5)*	*2 (3)*	*1 (3)*	*1 (3)*	*2 (7)*
Prior ASA (n, [%])	*13 (4)*	*2 (3)*	*2 (7)*	*1 (3)*	*0*
Chronic lung disease (n, [%])	*40 (12)*	*9 (13)*	*3 (10)*	*5 (14)*	*3 (11)*
Extracardiac arteriopathy (n, [%])	*15 (4)*	*4 (6)*	*1 (3)*	*1 (3)*	*1 (4)*
Neurologic dysfunction (n, [%])	*7 (2)*	*1 (1)*	*0*	*0*	*1 (4)*
Atrial fibrillation history (n, [%])	*65 (19)*	*8 (11)*	*1 (3)*	*4 (11)*	*4 (15)*
ICD history (n, [%])	*22 (7)*	*5 (7)*	*2 (7)*	*3 (8)*	*3 (11)*
Pacemaker history (n, [%])	*9 (3)*	*0*	*1 (3)*	*2 (6)*	*0*
LBBB (n, [%])	*33 (10)*	*5 (7)*	*0*	*6 (17)*	*1 (4)*
RBBB (n, [%])	*24 (7)*	*3 (4)*	*1 (3)*	*1 (3)*	*0*
Emergency operation (n, [%])	*5 (2)*	*0*	*1 (3)*	*0*	*2 (7)*

Endocarditis (n, [%])	*3 (9)*	*0*	*0*	*0*	*1 (4)*
*Pathogenetic DNA variants * & **(n, [%])*	*87 (58)*	*26 (62)*	*8 (27)*	*16 (44)*	*6 (22)*
Family history of HCM (first-degree relative)	*55 (16)*	*22 (31)*	*4 (13)*	*11 (31)*	*1 (4)*
NYHA functional class (n, [%])					
I & II	*131 (39)*	*32 (44)*	*9 (30)*	*6 (17)*	*7 (26)*
III & IV	*204 (61)*	*30 (66)*	*21 (70)*	*30 (83)*	*20 (74)*
Echocardiographic					
Systolic LVF (n, [%])					
Good (EF ≥ 50%)	*326 (97)*	*71 (99)*	*30 (100)*	*36 (100)*	*25 (93)*
Impaired (EF 40%–50%)	*8 (2)*	*1 (1)*	*0*	*0*	*2 (7)*
Moderately reduced (EF 30%–40%)	*1 (0)*	*0*	*0*	*0*	*0*
LAVI in mL/m² (median, [IQR])	*45 [37–55]*	*44 [33–59]*	*42 [37–49]*	*43 [35–48]*	*50 [41–55]*
LADI in mm/m² (median, [IQR])	*21 [19–24]*	*21 [18–23]*	*20 [18–24]*	*20 [18–23]*	*21 [20–22]*
Resting LVOT gradient (median, [IQR])	*60 [38–81]*	*9 [6–15]*	*50 [39–70]*	*53 [41–72]*	*66 [56–81]*
*Provocative LVOT gradient (mean, [SD])***	*85 (28)*	*93 (29)*	*84 (29)*	*73 (27)*	*95 (19)*
Mitral regurgitation (n, [%])					
Grade 1 + 2	*231 (69)*	*58 (81)*	*21 (70)*	*24 (67)*	*10 (37)*
Grade 3 + 4	*103 (31)*	*56 (32)*	*9 (30)*	*12 (33)*	*17 (63)*
Valvular SAM (n (%))	*268 (80)*	*65 (90)*	*28 (93)*	*25 (93)*	*25 (93)*
IVSd in mm (median, [IQR])	*21 [18–24]*	*12 [9–13]*	*20 [18–24]*	*21 [18–25]*	*12 [10–13]*
TAPSE in mm (median, [IQR])	*20 [20–24]*	*17 [14–18]*	*21 [20–25]*	*20 [20–23]*	*17 [16–18]*
Medication (n, [%])					
ACE-inhibitors	*59 (17)*	*7 (10)*	*3 (10)*	*5 (14)*	*5 (19)*

	Anticoagulation	*154 (46)*	*21 (29)*	*9 (30)*	*7 (19)*	*11 (41)*
	Amiodaron	*9 (2)*	*1 (1)*	*0*	*0*	*2 (7)*
	ARB	*65 (19)*	*9 (13)*	*4 (13)*	*5 (14)*	*6 (22)*
	B-blockers	*249 (74)*	*54 (75)*	*24 (80)*	*27 (75)*	*17 (63)*
	Calcium channel blockers	*107 (32)*	*28 (39)*	*13 (43)*	*13 (36)*	*9 (33)*
	Disopyramide	*16 (5)*	*3 (4)*	*0*	*4 (11)*	*1 (4)*
	Diuretics	*86 (26)*	*10 (14)*	*7 (23)*	*7 (19)*	*6 (22)*

*Values are mean ± standard deviation (SD), median [IQR], or n (%). Abbreviations: ACE, angiotensin-converting enzyme; ARB, angiotensin II receptor blocker; ASA, alcohol septal ablation; BMI, body mass index; ICD, implantable cardioverter defibrillator; IQR, interquartile range; IVSD, interventricular septal thickness in diastole; LADI, left atrial dimension index; LAVI, left atrial volume index; LBBB, left bundle branch block; LVF, left ventricular function; LVOT, left ventricular outflow tract; MM = millimetre; NYHA, New York Heart Association; RBBB, right bundle branch block; SAM, systolic anterior motion; SD, standard deviation; TAPSE, tricuspid annular plane systolic excursion. *more than 50% missing and therefore calculated patients with outcome, ** proportion of genotype-positive patients of genetically tested group.*

Table 11. Intraoperative and 30-day clinical outcomes in patients operated on with surgical myectomy in the Netherlands stratified by mitral valve surgery.

Characteristics	Overall (n = 335)	Isolated surgical myectomy (n = 72)	Surgical myectomy with E2E (n = 30)	Surgical myectomy with AMLE (n = 36)	Surgical myectomy with MV replacement (n = 27)
Intraoperative					
ACC time in minutes (median, [IQR])	*74 [46–126]*	*40 [28–58]*	*49 [38–68]*	*105 [72–134]*	*140 [112–210]*
CPB time in minutes (median, [IQR])	*113 [75–142]*	*63 [52–94]*	*82 [68–119]*	*150 [95–177]*	*209 [172–288]*
*VSD (n, [%])**	*7 (2)*	*1 (1)*	*1 (3)*	*0*	*1 (3)*
Echocardiographic (first after surgical myectomy)					
Systolic LVF (n, [%])					
Good (EF ≥ 50%)	*286 (85)*	*65 (90)*	*24 (80)*	*33 (9)*	*25 (78)*
Impaired (EF 40%–50%)	*37 (11)*	*6 (8)*	*6 (20)*	*3 (8)*	*2 (6)*
Moderately reduced (EF 30%–40%)	*0*	*0*	*0*	*0*	*0*
LAVI in mL/m^2 (mean, [SD])	*44 [34–55]*	*44 [33–59]*	*40 [35–46]*	*45 [35–52]*	*50 [41–55]*
LADI in mm (mean, [SD])	*22 [18–25]*	*21 [18–23]*	*18 [16–23]*	*21 [17–23]*	*21 [20–22]*
Resting LVOT gradient in mmHg (mean, [SD])	*9 [6–15]*	*9 [6–15]*	*14 [8–19]*	*11 [5–15]*	*7 [6–10]*
*Provocative LVOT gradient in mmHg (mean, [SD])***	*17 (16)*	*19 (17)*	*16 (2)*	*16 (9)*	*22 (22)*
Residual LVOT gradient					
Resting (≥ 30 mmHg) (n, [%])	*24 (7)*	*8 (11)*	*1 (3)*	*2 (6)*	*0*
*Provocative (≥ 50 mmHg) (n, [%]) *****	*3 (1)*	*0*	*0*	*0*	*0*
Mitral regurgitation (n, [%])					
Grade 1 + 2	*304 (90)*	*69 (72)*	*27 (90)*	*35 (97)*	*22 (82)*

		Grade 3 + 4	*18 (5)*	*3 (4)*	*3 (10)*	*1 (3)*	*0*
	Valvular SAM (n [%])		*28 (8)*	*9 (13)*	*4 (13)*	*3 (8)*	*0*
	IVSd in mm (median, [IQR])		*12 [10–14]*	*12 [9–13]*	*12 [10–16]*	*13 [11–13]*	*12 [10–13]*
	TAPSE in mm (mean, [SD])		*17 [14–18]*	*17 [14–18]*	*17 [14–18]*	*18 [15–20]*	*17 [16–18]*
Postoperative (either at 30-day or during hospital admission), composite endpoint (11%,; n = 38)							
	*Mortality (n, [%])**		*16 (5)*	*0*	*1 (3)*	*0*	*5 (19)*
	*Stroke (n, [%])**		*11 (3)*	*0*	*0*	*2 (6)*	*4 (15)*
	*Surgical reoperation (n, [%])**						
		Mitral valve replacement	*2 (1)*	*0*	*0*	*1 (3)*	*0*
		Mitral valve repair	*3 (1)*	*1 (1)*	*0*	*1 (3)*	*0*
		Myectomy	*3 (1)*	*1 (1)*	*0*	*1 (3)*	*0*
	New pacemaker (n, [%])		*34 (10)*	*8 (12)*	*3 (10)*	*2 (6)*	*1 (3)*
	New ICD (n, [%])		*12 (4)*	*5 (7)*	*0*	*0*	*1 (3)*
	Heart rhythm complications (n, [%])		*146 (44)*	*30 (42)*	*13 (43)*	*19 (53)*	*15 (47)*
	LBBB (n, [%])		*227 (68)*	*54 (75)*	*19 (63)*	*32 (89)*	*22 (74)*
	Mediastinitis (n, [%])		*4 (1)*	*0*	*0*	*1 (3)*	*0*
	Bleeding (n, [%])		*32 (10)*	*4 (10)*	*1 (3)*	*4 (11)*	*4 (15)*
	Hospital stay in days (median, [IQR])		*7 [6–12]*	*7 [5–8]*	*8 [6–14]*	*7 [6–9]*	*9 [5–13]*

*Values are mean ± standard deviation (SD), median [IQR] or n (%). Abbreviations: ACC, aortic cross-clamp; AVR, aortic valve replacement; CABG, coronary artery bypass grafting; CPB, cardiopulmonary bypass; ICD, implantable cardioverter defibrillator; IQR, interquartile range; IVSD, interventricular septal thickness in diastole; LADI, left atrial dimension index (mm/m^2); LAVI, left atrial volume index (mL/m^2); LBBB, left bundle branch block; LVF, left ventricular function;; SAM, systolic anterior motion; TAPSE, tricuspid annular plane systolic excursion; TVR, tricuspid valve replacement; VSD, ventricular septal defect. *Composite endpoint occurred in 11% of patients (n = 38) , **more than 50% missing and therefore calculated patients with outcome, **** patients experienced also residual resting LVOT obstruction (≥ 30 mmHg).*

Supplemental Figures

Supplementary Figure S1. Typical data collection timepoints

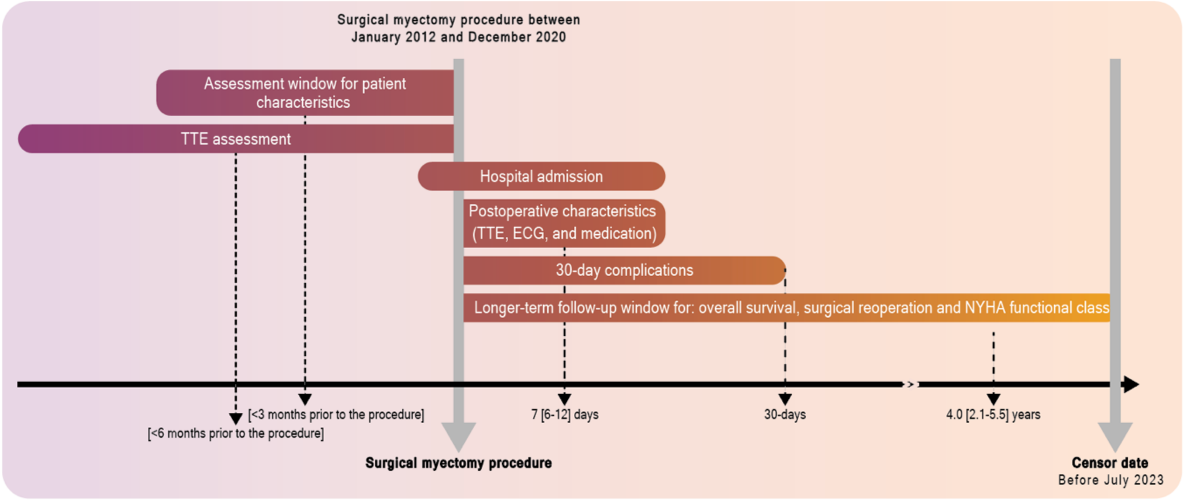

Timepoints are presented in median (interquartile range [IQR]) in days or median [IQR] in years.

Supplementary Figure S2. Flowchart demonstrating a selection of HOCM patients operated on with surgical myectomy.

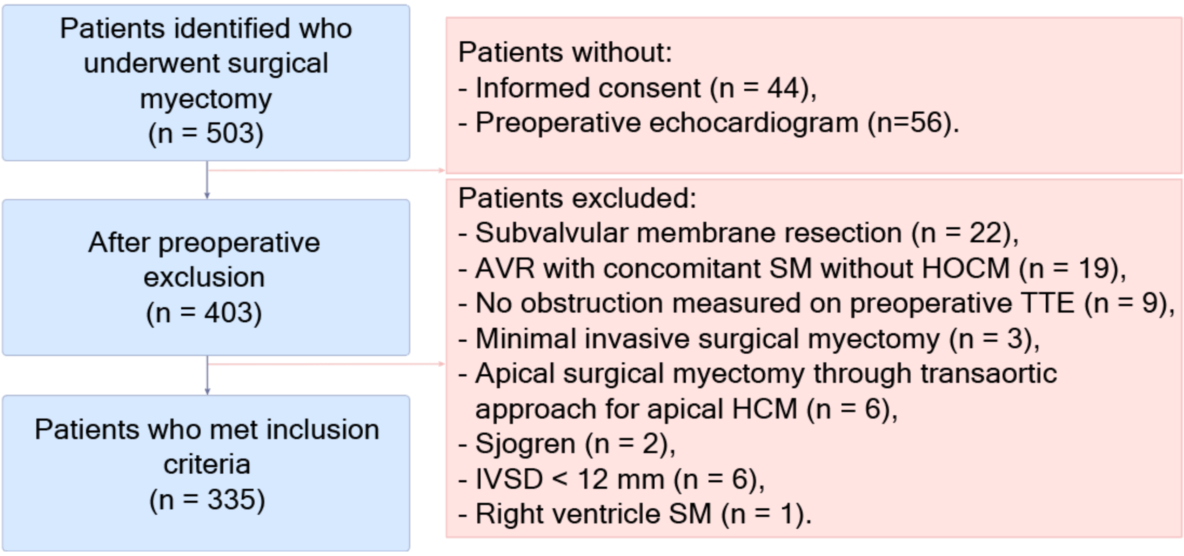

Abbreviations: AVR, aortic valve replacement; HOCM, hypertrophic obstructive cardiomyopathy; IVSD, interventricular septal thickness in diastole; SM, surgical myectomy; TTE, transthoracic echocardiogram.

Supplementary Figure S3. Number of surgical myectomy with concomitant mitral valve repair techniques in the Netherlands stratified for intrinsic mitral valve disease.

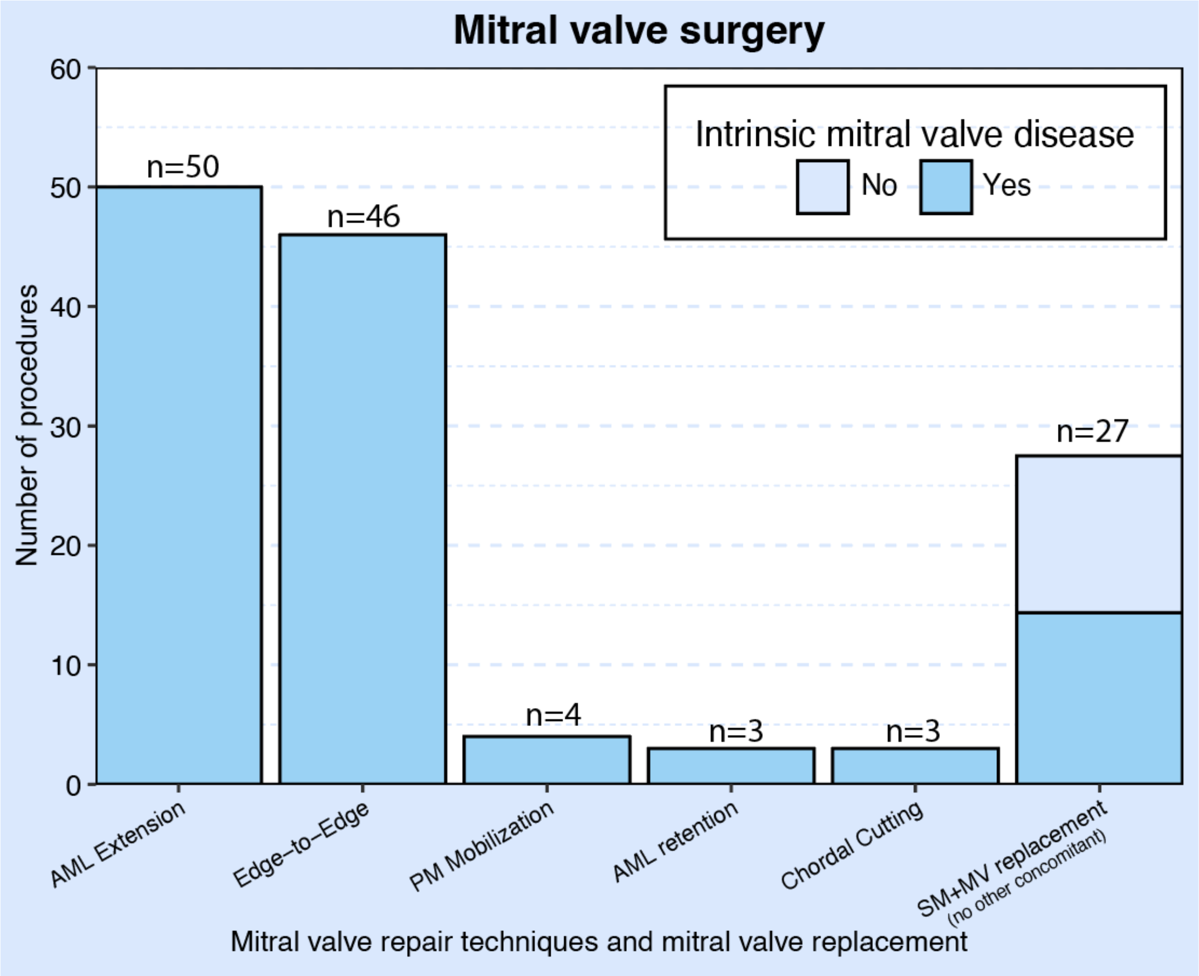

The different concomitant mitral valve repair techniques and mitral valve replacements are presented. The stacked bars represent the number of intrinsic mitral valve disease for concomitant mitral valve replacement. Abbreviations: AML, anterior mitral valve leaflet; MV, mitral valve; PM, papillary muscle mobilization; SM, surgical myectomy.

Supplementary Figure S4. Kaplan-Meier plot for overall survival among patients with hypertrophic obstructive cardiomyopathy patients undergoing underwent surgical myectomy in the Netherlands.

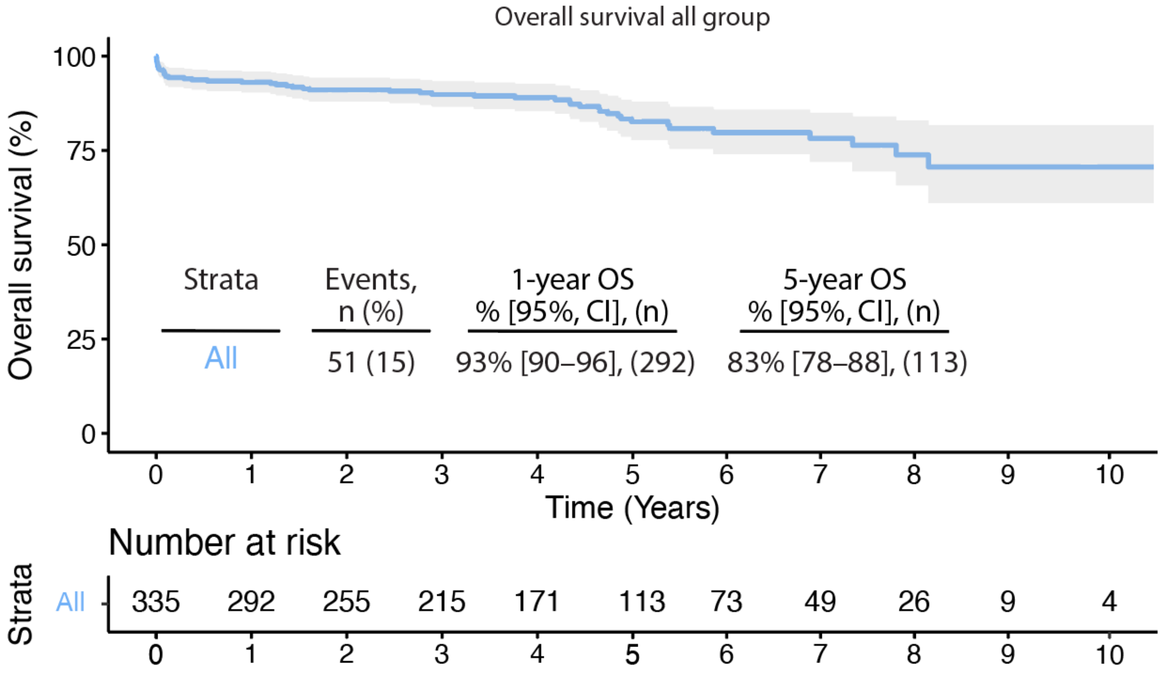

Abbreviations: OS, overall survival.
